# When the killing has been done: Exploring associations of personality with third-party judgment and punishment of homicides in moral dilemma scenarios

**DOI:** 10.1371/journal.pone.0235253

**Published:** 2020-06-30

**Authors:** Alexander Behnke, Anja Strobel, Diana Armbruster

**Affiliations:** 1 Faculty of Psychology, Technische Universität Dresden, Dresden, Germany; 2 Department of Psychology, Chemnitz University of Technology, Chemnitz, Germany; Middlesex University, UNITED KINGDOM

## Abstract

Killing people is universally considered reprehensible and evokes in observers a need to punish perpetrators. Here, we explored how observers’ personality is associated with their cognitive, emotional, and punishing reactions towards perpetrators using data from 1,004 participants who responded to a set of fifteen third-party perspective moral dilemmas. Among those, four scenarios (*architect*, *life boat*, *footbridge*, *smother for dollars*) describing deliberate killings were compared to investigate the role of the content features “motive for killing” (selfish vs. utilitarian) and “evitability of victims’ death”. Participants’ moral appropriateness ratings, emotions towards perpetrators, and assigned punishments revealed complex scenario-personality interactions. Trait psychopathy was associated with harsher punishments in all scenarios but also with less concern about killing in general, an increased moral appreciation of utilitarian motives for killing, and a reduced concern about the killing of avoidable victims. Need for cognition was associated with considering a utilitarian motive for killing as a mitigating factor, while intuitive/authority-obedient thinking was linked to a strong focus on avoidability of harm as an aggravating factor when assigning punishments. Other-oriented empathy, trait anxiety, and justice sensitivity did not account for differences in third-party punishments. Our explorative findings highlight the importance of inter-individual differences for moral decision making and sense of justice.

## 1. Introduction

Monitoring compliance with rules of social coexistence and punishing their violation are essential for the stability of human societies. Especially in growing and increasingly anonymous societies, uninvolved third parties play a central role in restoring and enforcing rule compliance [1,2]. When observing violations of socio-moral rules resulting in negative consequences for victims, third parties typically sense a strong obligation to punish perpetrators and/or try to compensate victims in order to restore justice. This “altruistic” motivation to punish arises although observers themselves are not negatively affected and although they have no direct benefit from punishing perpetrators [3,4]. In fact, the evaluation of rule transgressions by uninvolved third parties (e.g., neutral judges or lay assessors) is a basic principle of most legal systems.

When evaluating rule transgressions, third parties may take into account the intentions or goals of perpetrators as well as possible situational constraints under which perpetrators acted [5,6]. However, due to differences in available information or lack of personal consequences the perception of justice and moral appropriateness may vary considerably with different social perspectives [7,8]. There are numerous potential modulators that might affect whether third parties judge a rule transgression as inappropriate and how they punish agents and/or try to compensate victims [6,9,10]. In this study, we investigate whether and how personality traits account for inter-individual differences in observers’ responses towards acts of killing. We explore how personality traits previously associated with moral decision making, moderate i) emotional reactions of observers, ii) their assessment of moral appropriateness, and iii) the severity of imposed punishments. In short, we explore whether personality traits are associated with differences in observers’ tendencies to take avoidability of victims’ death and perpetrators’ motives for killing into account.

### 1.1 Thou shalt not kill

The condemnation of killing as stated in, for instance, the 5^th^ commandment (God, in Exodus 20:13, 6^th^ century BC), is one of the most important moral principles in basically all human societies [11,12]. Although this moral imperative clearly forbids the murder of fellow human beings, exceptions to this rule exist. Various reasons have been brought forward to justify killing others. Examples include self-defense, capital punishment, or warfare. Under these circumstances, observers might judge acts of killing as justifiable and therefore punish perpetrators less severely or not at all.

Moral dilemmas allow to experimentally investigate how different situational characteristics determine whether the killing of another person is deemed morally permissible and therefore not (or less) punishable [13,14]. The hypothetical short stories describe scenarios with two mutually exclusive outcomes, both violating essential moral principles [15–17]. Moral dilemmas have been predominantly used to investigate decisions from the first-party perspective, i.e., under which circumstances participants would decide to kill another person [16,18–21]. Various situational features were identified to influence moral decision making, including the use of personal force as well as the evitability and intentionality of harm [13,22,23]. There are considerably less studies on the *third-party perspective*, in which participants judge whether the choice of another person is morally adequate. Some studies reported different moral responses in first vs. third parties [24,25], while others observed no differences [16,26].

In this study, we explore potential effects of personality as well as dilemma content features that were previously shown to be of relevance for third-party judgments. Perceived moral reprehensibility of active killing of one or a few individuals (as opposed to death due to neglect) varies, for instance, due to the following two features [cf. 22]: (a) *motive for killing* (selfishness vs. utilitarian motive, i.e., saving multiple people by sacrificing one or a few) [24–26], and (b) *inevitability* of the victims’ death [27].

From the perspective of moral decision making, the aforementioned 5^th^ commandment constitutes a *deontic* rule. Such rules define fundamental principles of right and wrong actions (or inactions) independent of their effect on the “greater good”. In contrast, *utilitarian* (or consequentialist) principles focus on the consequences of these actions. Both principles have been proposed to vie for influence when making moral decisions [13,15,17]. Depending on the situation, they are not necessarily in conflict with each other [28]. However, in the case of killing for self-interest, the deontic rule is violated without producing a benefit for the majority. Thus, killing for self-interest presents a *congruent* rule transgression since it also violates utilitarian principles [28]. However, the killing of a small number of individuals to save a larger group brings deontic and utilitarian rules in conflict and represents an *incongruent* rule transgression. If observers conclude that perpetrators acted for utilitarian reasons, they can generally be expected to judge the decision as more morally appropriate [24–26] and to punish perpetrators less severely [26]. It was the first aim of our study to *explore* whether personality traits are associated with differences in observers’ tendency to take perpetrators’ utilitarian motives into account.

In addition, observers are likely to take circumstances into account which result in the *inevitable* death of victims. In such situations, the utilitarian option might indeed offer an acceptable solution even if deontic rules are violated: Try to save as many people as possible while sacrificing as few as possible instead of refraining from intervening altogether which results in the death of *all* [14]. Previous studies reported that observers consider harmful utilitarian actions more justifiable when harm to the victim is inevitable anyway [27]. Thus, observers are expected to punish killings less severely if the circumstances made the victims’ death inevitable. Therefore, our second aim was to *explore* possible associations between personality traits and third-parties’ tendencies to take the (in-)evitability of victims’ death into account when judging and punishing homicides.

Moral judgments involve a complex interplay of cognitive processes and emotional reactions [20,23,29]. While some authors highlight the role of deliberate cognitions, others put greater emphasis on intuitive emotional tendencies (*moral sentiments*; for a review see [23]). Third parties punish more harshly if the immoral behavior is deemed more reprehensible and when greater harm or unfairness is inflicted on victims. Third parties also report stronger negative emotions towards perpetrators like anger, contempt, disgust or disappointment [3,5,29–32]. In turn, stronger negative emotions facilitate harsher punishment [3,5,29,33–35], indicating that the effect of observed harm or unfairness on third-party punishment could be mediated by negative emotions [36–38]. So far, most studies focused on single, predominantly negative emotions. However, ambiguous situations result in a complex mixture of partly conflicting emotions including positive notions like sympathy, comprehension, or compassion [31,39–41]. Positive emotions towards perpetrators have been reported to result in weakened negative emotions and milder punishment [36,42,43].

In sum, observers are expected to show a clear pattern of intensive negative emotions, but hardly understanding emotions towards perpetrators who kill for selfish reasons. Observers are also expected to judge such actions as more morally inappropriate and to severely punish them. In contrast, in cases of utilitarian killings and killings of inevitably dying victims, observers are expected to feel negative *and* understanding emotions towards the perpetrators. This ambiguous emotional state is expected to be accompanied by less clear judgements of moral appropriateness and eventually, milder punishments. Aside from these general assumptions, we explored whether third-parties’ emotional, cognitive, and punitive reactions were modulated by their personality.

### 1.2 Personality and moral decision making

Various personality traits have been associated with justice perception, helping behavior, and moral decision making. *Empathy* enables people to share the affective state of victims. To morally evaluate hypothetical actions, participants put themselves at the receiving end of the action (cognitive empathy) and experience what victims would experience (affective empathy) [44,45]. In moral dilemmas, empathic individuals may develop a strong emotional discomfort by being more immersed in the position of threatened individuals as well as by imagining the harmful act itself [46]. In previous studies on moral decision making from the first-party perspective, more unpleasant feelings during decision making were linked to higher scores of *other-oriented empathy*, particularly empathic concern and perspective taking. In general, empathic concern is linked to less utilitarian and more deontic decisions [28,47–50]. However, Reynolds and Conway [51] showed that empathic concern specifically predicts a stronger inclination to deontic choices, but was *not* associated with utilitarian tendencies. So far, empathy has not been investigated in the context of third-party punishments in moral dilemmas.

Experiencing warmth, compassion, and concern for others in need is considered to promote a true *altruistic* motivation with the final goal to increase others’ welfare. Since its introduction in the 19^th^ century, the definition of *altruism* has undergone several modifications [52]. Broadly, it refers to the tendency to place the needs of others above one’s own. Altruism was suggested to induce uninvolved third parties to put effort in costly punishments of moral transgressions with no or little direct benefit for those who punish [53]. However, trait altruism has not yet been investigated in the context of third-party punishment.

Adopting a victim’s perspective can cause emotional distress in third parties by empathizing with victims’ suffering or by imagining perpetrators performing harmful actions [54,55]. Individuals scoring high in the empathy sub-trait *personal distress* as well as trait anxiety (i.e., *neuroticism*) tend to feel anxious and uncomfortable in tense interpersonal settings and report lower self-esteem [56,57]. Moreover, when confronted with others’ suffering, they are more likely to adopt an emotional self-focus (*self-oriented empathy*), characterized by feelings of fear, discomfort, and shame. To end their own negative empathic affect, these individuals tend to help quickly or−even more often−to evade the situation resulting in less prosocial behavior [31,55,56]. In first-party-perspective moral scenarios, *personal distress* was associated with less utilitarian decisions [55]. Individuals experiencing empathic distress might refrain from thinking longer about moral dilemmas to avoid negative feelings caused by the suffering of others. As a result, they quickly chose deontic solutions when making moral judgements [55]. Furthermore, trait *anxiety* was hypothesized to promote avoidance of (first-party) utilitarian decisions and to facilitate retributive punishment of non-deontic actions, but the existing evidence is inconsistent [58–60].

In research on social rule transgressions, *psychopathy* is one of the most prominent influential factors. Characteristic sub-traits of psychopathy include lack of remorse or guilt, shallow affect, callousness, manipulative tendencies, and antisocial behavior [61–63]. Psychopaths’ deviant behavior was linked to deficits in judging the permissibility of actions in moral dilemmas [63–65]. It was also associated with caring less about enforcing moral norms, especially fairness and harm prevention [66–68]. Other studies reported that psychopaths classified utilitarian actions as immoral, but nevertheless consistently preferred them [69–71]. However, moral dilemma studies applying process-dissociation approaches showed that while trait psychopathy is associated with a reduced inclination to deontic decisions, it is *not* associated with heightened tendencies for utilitarian decisions [51,72]. Individuals with higher trait psychopathy are less sensitive to moral norms as well as the consequences of their decisions and also less reluctant to act in general [73]. One explanation for their reduced endorsement of deontic principles could be their lower empathic concern for potential victims or individuals in need [49,51,61,66,67,74–76]. Previous studies found that disrespect for deontic rules is accompanied by reduced aversive emotional responses to witnessing or inflicting harm [63]. Absence of empathic concern for others goes along with less moral-reconstituting anger in observers when witnessing unfair treatment of others [30]. Correspondingly, individuals with higher trait psychopathy report lower reactive aggression and retaliation for rule violations appears to be less important to them [59,77]. Rather, such individuals seem to show increased proactive aggression as a means of deterrence. Thus, they are particularly prone to severely punish perpetrators who are likely to repeat rule transgressions [59,77]. Interestingly, in economic games, individuals with higher trait psychopathy punish other players who seek to take unfair advantages harshly, while at the same time they try themselves to cheat [78,79]. Based on these findings, one might expect that third parties with higher trait psychopathy tend to judge homicides as less morally inappropriate while feeling less negative emotions as well as more understanding emotions towards perpetrators. However, it is unclear whether this translates to reduced punishments.

An additional relevant concept is *justice sensitivity*, which refers to the perception of injustice or unfairness from different social perspectives, i.e., victim, perpetrator, observer, or beneficiary [7]. Observer and perpetrator justice sensitivity were consistently associated with fairness behavior (i.e., *other-oriented justice sensitivity*) [80]. Individuals with higher other-oriented justice sensitivity rated perceived immoral behavior in moral dilemmas as less permissible and showed more empathic concern and perspective taking as well as lower trait psychopathy [62]. Moreover, observer justice sensitivity was associated with increased third-party punishment and accompanying feelings of anger [81]. Conversely, victim sensitivity was linked to egocentric justice concerns and ignoring unfairness towards others [80].

Furthermore, thinking styles like *need for cognition* (NFC) and *faith in intuition* (FI) [82] were reported to influence moral behavior [83]. Deliberate thinking styles (e.g., NFC) were positively associated with utilitarian judgments in moral scenarios [28,84] as well as endorsement of different aspects of moral attitudes or behavior beyond moral dilemmas [83,85–87]. Importantly, individuals with deliberate thinking styles appear to prefer utilitarian options because of more marked concerns about optimizing overall welfare, not because they do not care about the deontic principle of preventing instrumental harm (as seen in psychopathic individuals) [28,88]. Correspondingly, experimental priming of deliberate thinking specifically increased utilitarian inclinations but did *not* influence deontic tendencies in moral decision making [89]. Furthermore, priming intuitive thinking does not diminish utilitarian tendencies per se but differentially affects two key components (instrumental harm vs. impartial beneficence [cf. 90]). Specifically, it decreases endorsement of instrumental harm but does not affect commitment to impartial beneficence [90]. However, the state of research concerning intuitive thinking is less conclusive. Bartels [91] reported that a factor including faith in intuition and NFC influenced participants’ inclination for deontic and utilitarian decisions, respectively. However, other studies could not establish a connection between faith in intuition and moral behavior [28,83]. Regarding punishment, Sargent [92] observed less support for punitive responses to crime in individuals with higher NFC scores. The association was mediated by attributional complexity, suggesting that high-NFC individuals take the complexity of perpetrators’ motives, reasons, and situational constraints more into account when judging behavior. Concordantly, cognitively exhausted participants were more susceptible to framing biases when responding to moral dilemmas with harmful utilitarian actions. In general, cognitive exhaustion led to less endorsement of utilitarian decisions [28,93]. Thus, if observers are un*able* or un*willing* to mobilize sufficient cognitive effort to deliberate perpetrators’ situation and motives, they tend to rely on situationally invariant deontic judgment templates [92].

Similarly, individuals with *authority-obedient* mindsets are less likely to consider more than one perspective on a (moral) problem, especially when it comes to questions concerning existential issues such as life and death [94]. Rather they tend to “solve” moral problems through a quick and situationally invariant application of deontic moral rules [11,28,51,94–96]. In general, authority-obedient individuals tend to feel more pronounced aggressive feelings towards violators of deontic norms and impose harsher punishments on them [97]. Thus, they might tend to predominantly focus on deontic rule transgressions when judging moral decisions.

In sum, based on the body of literature, the following traits were investigated for associations with third-party judgements: *other-* and *self-oriented empathy*, *altruism*, trait *anxiety* and *self-esteem*, trait *psychopathy*, *justice sensitivity*, *obedience to authorities* as well as the thinking styles *NFC* and *faith in intuition*. Several of them have conceptual similarities and were reported to systematically correlate. Thus, we extracted higher-order personality factors from these traits to reduce complexity and enhance comprehensibility of the analyses and the interpretation of their results. To the best of our knowledge, this is the first study to investigate associations of a broad set of personality traits with third-party judgment of acts of killing. This study is of explorative nature and aimed at examining the interplay between scenario characteristics (i.e., utilitarian motive to kill, preventability of victims’ death) and personality differences with regard to third parties’ cognitive, emotional, and punitive responses. Our results might thus serve as a basis for specific hypotheses to guide future research.

## 2. Methods

### 2.1 Participants

Complete data was available from *N* = 1004 participants (534 women, 470 men) mainly aged between 22 and 27 years (*Mdn* = 24.5, *IQR* = 4.6, range 18.1–58.0). The sample was primarily comprised of native German speakers (97.6%). The majority were high-school graduates (99.1% achieved the German Abitur) who were either currently enrolled as university students (62.2%) or had already graduated from university (31.3% with Diploma or Master’s degree). The remaining participants (5.9%) were undergoing vocational job trainings. The sample included all academic disciplines, with a low proportion of psychology students (7.1%). With regard to religious belief, 52.8% considered themselves atheist or agnostic, 37.1% Christian (including 23.8% Protestants and 12.4% Catholics) and 6.9% as adhering to other beliefs, mainly Buddhism and natural religions.

### 2.2 Procedure

This study was conducted as part of a broader research project. We report how we determined our sample size, all data exclusions, all manipulations, and all measures in the study [98]. The study concept was approved by the Technische Universität Dresden ethics committee (proposal no. EK241062016). Since effects of personality traits are generally rather small, we aimed at recruiting about 1,000 participants. Participants were recruited via social media announcements and invitation emails in distribution lists of several German-speaking European universities and the German National Academic Scholarship Foundation. A link took interested participants to a two-stage online survey [99]. Before entering the survey, participants were informed about general study procedures and aims and indicated their informed consent by clicking a checkbox. In the first survey session, *n* = 1,563 participants completed sociodemographic and personality questionnaires. One week later, they received an invitation to the second session, during which they read short moral scenarios phrased in the third-party perspective. Participants rated their emotional responses and judged the behavior of the scenarios’ protagonists. A total of *n* = 1,004 participants completed the second survey. No participants were excluded. The entire survey took between 30–60 minutes to complete. All scenarios included in this study, a list of questionnaires used, measures of emotional, cognitive, and punitive responses, as well as data and code are openly available [100].

### 2.3 Experimental design

We investigated the interplay of the two content factors, (1) motive for killing (selfish vs. utilitarian) and, (2) avoidability of victims’ death (avoidable vs. inevitable) with third parties’ personality. For this purpose, we chose to compare four scenarios which, among other factors, differ regarding the two content factors and rephrased them in the third-party perspective: *lifeboat*, *footbridge*, *smother for dollars*, and *architect* [101,102] (see S1 File).

Briefly, in the (i) *architect* scenario, the killing is done out of pure selfish reasons: An architect pushed his boss from a scaffold to his death because he had been repeatedly treated rudely by him. (ii) In the *smother for dollars* scenario, the protagonist, instigated by the son of a terminally ill man, suffocates the patient to enrich himself on the patient’s life insurance. (iii) In the *footbridge* scenario, the protagonist pushed a large stranger from a footbridge on a railway track to stop an approaching trolley that otherwise would have killed five railway workers. (iv) In the *lifeboat* scenario, crew and passengers of a sinking ship got into lifeboats. However, due to overcrowding and rough waves the lifeboats are about to sink as well, which would result in the death of practically everyone. Therefore, a senior officer decided to push some people overboard so that at least the remaining ones might survive.

The scenarios *architect* and *smother for dollars* describe homicides that simultaneously violate deontic *and* utilitarian rules (i.e., a *congruent* transgression [28]). In contrast, *footbridge* and *lifeboat* present *incongruent* scenarios [28], in which the two moral principles imply different courses of action. The chosen actions follow the utilitarian principle to minimize net harm while at the same time violating the deontic rule not to kill. We compared selfish and utilitarian killings to investigate the hypothesis that third parties take perpetrators’ *utilitarian motive* into account as mitigating factor.

In the scenarios *architect* and *footbridge*, the death of the victims is entirely preventable, whereas in *smother for dollars* and *lifeboat*, the death of the victims is inevitable regardless of the chosen action. Killing avoidable and inevitable victims was compared to investigate whether third parties consider the *inevitability* of the death of those killed as a mitigating factor when assigning punishment and making moral judgments [27].

In the course of the revision process, the analysis strategy was modified and extended. Originally, three dilemmas (architect, footbridge, lifeboat) were used to analyze the role of the two target content features. To investigate ‘motive for killing’ footbridge and architect were contrasted, while ‘avoidability of victims’ death’ was investigated by contrasting footbridge and lifeboat. The original analysis and its results can be accessed at https://osf.io/3wsxh/. The original findings were confirmed by the new analysis.

All participants read and responded to all scenarios providing *within*-subject comparisons. After an exercise scenario familiarizing participants with the procedure and the input mask for their responses, fifteen scenarios were presented. Among them, seven scenarios described instrumental killings, including the four scenarios examined in this study, while eight scenarios described harmful omissions, including two scenarios addressing another research question on utilitarian rule transgressions [103]. To avoid carry-over effects [104], the four scenarios of interest were not presented in direct succession but were embedded in a randomly chosen scenario series (see S1 File).

### 2.4 Supplementary scenario contrasts

In addition to the main analysis we conducted supplementary analyses to determine whether the explored personality effects hold up across different scenarios involving instrumental killings. Due to the high content heterogeneity of the moral dilemmas used (see S1 File), we refrained from simply aggregating responses across scenarios to avoid unwanted error variance [22]. Based on dilemmas’ content features, we limited the supplementary analyses to the following additional contrasts: (1) the *footbridge* scenario was contrasted with the *transplant* scenario. In the latter, a physician kills a healthy patient and transplants his organs to save five other patients. Both scenarios describe an instrumental killing of an avoidable victim but differ with regard to the relationship between perpetrator and victim as well as the physician’s additional professional duty to do no harm. (2) We contrasted the scenarios *crying baby* and *Sophie’s choice*, both describing how a mother decides—in order to save another family member—either to kill her own child by suffocating it (*crying baby*) or to hand it over to be killed by others (*Sophie’s choice*). This contrast allows for an investigation of the extent to which observers take personal vs. impersonal killing into account.

### 2.5 Assessment of participants’ responses

After reading each scenario, participants responded to several rating scales assessing their emotional, cognitive, and punitive reactions to the scenario (see S1 File for an overview of all assessed variables). Participants rated the intensity of their emotions towards the scenario’s protagonist on 7-point Likert scales ranging from 0 (*not at all*) to 6 (*very intensive*). A total of seven emotional items was presented with the question “When I think of the protagonist and his/her decision, I feel (…)”. For later analysis, the four items (1) anger/outrage, (2) contempt, (3) moral disgust, and (4) disappointment were aggregated to negative/hostile emotions (Cronbach’s α = .88, .89, .89, and .91 in *architect*, *smother for dollars*, *footbridge*, and *lifeboat*, respectively). Similarly, the items (1) comprehensive affection, (2) sympathy, and (3) compassion/pity were combined in a scale of understanding emotions (Cronbach’s α = .84, .79, .83, and .81 in *architect*, *smother for dollars*, *footbridge*, and *lifeboat*, respectively). Participants rated the moral appropriateness of the described action on a 7-point Likert scale ranging from 0 (*completely disagree*) to 6 (*completely agree*). Finally, they decided whether and how long perpetrators should be imprisoned. Imprisonment length could be freely set with a slider within the limits of 0 to 100 years. Other types of punishment (e.g., fines, community work or death penalty) could not be imposed. Participants also rated whether it was difficult to reach a judgement in the respective scenarios on a 7-point Likert scale ranging from 0 (*completely disagree*) to 6 (*completely agree)*. There was no time limit for reading the scenarios or answering the items.

### 2.6 Assessment of personality

Personality traits were assessed using validated scales. To keep processing time for participants within reasonable limits, we applied—if available—short inventories and cleared inventories from scales not relevant for our research questions. *Trait psychopathy* was assessed with a German version of the Self-Report Psychopathy scale, version III (SRP-III) [105]. Three of SRP-III scales were administered, namely interpersonal manipulation, callous affect, and erratic lifestyle with 16 items each (Cronbach’s α = .79, .77, and .74, respectively). The fourth scale (antisocial behavior) was omitted because investigations in community samples revealed extremely low means and very little variance. Using the German Interpersonal Reactivity Index (IRI-SPF) [106], *other-oriented empathy* was assessed with the three scales fantasy, empathic concern, and perspective taking, whereas *self-oriented empathy* was assessed with the scale personal distress (Cronbach’s α = .73, .73, .75, and .67, respectively, with four items each). Note that the personal distress scale typically has a lower internal consistency [107]. *Altruism* was assessed using the respective scale of the German NEO Personality Inventory-Revised (NEO-PI-R [108]; Cronbach’s α = .73). The traits *need for cognition* (NFC) and *faith in intuition* were assessed according to the Cognitive-Experiential Self-Theory [82] using the German Rational-Experiential Inventory (REI [109]; Cronbach’s α = .84 and .85, respectively). *Justice sensitivity* [7] was assessed by administering the Injustice Sensitivity-Short Scales (USS-8) [110]. On four scales, the inventory differentiates participants’ inclination to experience injustice from the four social perspectives victim, perpetrator, beneficiary, and observer. Cronbach’s α could not be determined as each scale consisted of two items only. *Obedience to authorities* was measured using the two-item short scale of the ALLBUS 2012 Questionnaire [111]. *Trait anxiety* was assessed using the 12-item neuroticism scale of the German NEO-Five-Factor Inventory (NEO-FFI [112]; Cronbach’s α = .89) and *self-esteem* with the single-item self-esteem short scale [113].

### 2.7 Statistical analyses

Assessed personality traits covaried systematically due to conceptual overlaps. Thus, we extracted higher-order personality domains with an exploratory factor analysis (EFA). Data covariance was adequate for conducting a meaningful EFA (Kaiser-Meyer-Olkin measure of sampling adequacy = .770; Bartletts’s Sphericity test: χ^2^(136) = 5373.08, *p* < .001). A principal component analysis was conducted to maximize the extracted variance. Factors with Eigenvalues λ ≥ 1 were extracted and obliquely Promax-rotated (κ = 4, with Kaiser normalization).

Associations between personality factors and participants’ responses to the moral scenarios were calculated using bivariate nonparametric Kendall’s τ rank correlations. Correlations between measures repeatedly collected across the four scenarios (i.e., assigned imprisonment, moral emotions, moral appropriateness rating) were calculated as repeated measures correlations (using the R package *rmcorr* [114]).

Linear mixed effect models were conducted (using the R packages *lme4* and *lmerTest* [115,116]) to analyze main and interaction effects of the two content factors and the extracted personality factors. All models included (i) the content factors “motive for killing” (selfish vs. utilitarian) and “avoidability of victims’ death” (avoidable vs. inevitable) as well as their interaction as within-subject factors, (ii) the personality factors as between-subject factors, and (iii) all three-way interactions between the two content factors and one personality factor. Each dependent variable—i.e., imposed imprisonment, intensity of negative emotions, intensity of understanding emotions, and perceived moral appropriateness—was analyzed separately. Thus, *p*-values were Bonferroni-corrected for the number of outcome variables (α_*crit*_ < 0.0125). Post-hoc contrast tests were computed to characterize the nature of significant interaction effects (using the R package *emmeans* [117]). Due to computational limitations, asymptotic degrees of freedom were used. The family-wise error rate was controlled using a Holm correction of *p*-values.

## 3. Results

### 3.1 Exploratory factor analysis

The EFA assembled the 17 personality-trait facets under five superior personality factors, representing 63% of the traits’ variance (Table 1). The first personality factor (PF1) includes altruism, faith in intuition, victim injustice sensitivity, (less) callous affect and the other-oriented empathic traits fantasy, empathic concern, and perspective taking. The second factor (PF2) is characterized by increased neuroticism (i.e., trait anxiety) and personal distress with decreased self-esteem. The third factor (PF3) consists of increased trait psychopathy, with simultaneously lower altruism and increased justice sensitivity from the perpetrator’s perspective. The fourth factor (PF4) represents general justice sensitivity across all perspectives (i.e., perpetrator, victim, observer, beneficiary). The fifth factor (PF5) combines marked faith in intuition and little motivation for critical, analytical thinking (i.e., low NFC) with increased authority obedience and greater justice sensitivity from the victim perspective. PF1, PF3, and PF4 were moderately correlated.

**Table 1:** Results of exploratory factor analysis.

Entered Personality Facets	Extracted Personality Factor					Communalities *h*^2^
	*PF1*	*PF2*	*PF3*	*PF4*	*PF5*	
Altruism (NEO-PI-R)	**.478**	-.246	**-.441**	.000	.149	.651
Neuroticism (NEO-FFI)	.187	**.790**	.155	.159	.145	.759
Victim justice sensitivity (USS-8)	**-.340**	-.024	.094	**.665**	**.435**	.644
Observer justice sensitivity (USS-8)	.111	-.019	.022	**.793**	-.095	.683
Beneficiary justice sensitivity (USS-8)	.045	.117	-.021	**.707**	-.165	.576
Perpetrator justice sensitivity (USS-8)	.065	.020	**-.426**	**.436**	-.243	.564
Faith in intuition (REI)	**.458**	-.327	.193	.066	**.563**	.656
Need for cognition (REI)	.096	-.288	.145	.149	**-.757**	.696
Obedience to authorities	-.049	.141	-.141	-.125	**.694**	.509
Self-esteem	.002	**-.798**	.124	-.062	-.055	.706
Interpersonal manipulation (SRP-III)	.049	-.020	**.845**	.027	-.007	.678
Callous affect (SRP-III)	**-.410**	.023	**.605**	-.059	-.105	.751
Erratic life-style (SRP-III)	.261	-.073	**.762**	.047	-.176	.511
Fantasy (IRI-SPF)	**.807**	.234	.301	-.015	.020	.560
Empathic concern (IRI-SPF)	**.722**	.191	-.108	.138	-.012	.708
Perspective taking (IRI-SPF)	**.645**	.043	.025	-.083	-.282	.441
Personal distress (IRI-SPF)	.181	**.781**	-.047	-.090	.125	.628
	***PF1***	***PF2***	***PF3***	***PF4***	***PF5***	Total
Factor Eigenvalues (λ)	3.94	2.67	1.57	1.47	1.08	
Variance proportion (%) per factor	23.18	15.69	9.24	8.63	6.33	63.07
**Domain correlation matrix**	***PF1***	***PF2***	***PF3***	***PF4***	***PF5***	
PF1	1.00	-.11	-.37	.37	.02	
PF2		1.00	-.07	.20	.13	
PF3			1.00	-.23	.12	
PF4				1.00	.11	
PF5					1.00	

### 3.2 Descriptive statistics and correlations

Table 2 presents descriptive statistics and correlations of the outcome variables. Bivariate correlations between personality domains and outcome variables are displayed in Table 3. Across the scenarios *architect*, *smother for dollars*, *footbridge*, and *lifeboat*, there was a decrease in punishment and negative emotions, while comprehensive emotions and moral appropriateness increased. Participants reported the greatest difficulties to decide an appropriate level of punishment in the *footbridge* scenario. A detailed analysis (see S1 File) revealed that decision difficulty was a reverse parabolic function of the moral emotion conflict (S1 Fig in S1 File). Unsurprisingly, participants found it easier to judge a protagonist’s behavior when either negative or understanding emotions towards the perpetrator clearly dominated. Contrariwise, when both emotional tendencies were equally strong, decisions on appropriateness were harder to make.

**Table 2:** Descriptive statistics and correlations of outcome variables.

	Descriptive Statistics *M* (*SD*)				Repeated-measures correlations				
Outcome	Architect	Smother for dollars	Footbridge	Lifeboat	1.	2.	3.	4.	5.
1. Punishment (imprisonment in years)	24.67 (7.43)	15.80 (7.24)	5.58 (2.85)	3.94 (2.55)	1	.53***	-.73***	-.72***	-.49***
2. Negative Emotions	3.12 (1.05)	2.98 (1.24)	2.38 (1.02)	2.22 (0.86)		1	-.51***	-.51***	-.31***
3. Understanding Emotions	0.76 (0.98)	1.00 (1.21)	2.39 (1.33)	3.47 (1.31)			1	.82***	.38***
4. Appropriateness	0.19 (0.62)	0.63 (1.18)	1.77 (1.41)	2.85 (1.26)				1	.32***
5. Decision Difficulty	1.29 (1.56)	1.95 (1.75)	3.05 (2.06)	2.79 (2.01)					1

*** *p* < .0001, two-tailed, Bonferroni-corrected.

**Table 3:** Correlations of experimental outcomes and personality domains (*N* = 1004).

		***Architect***				***Smother for dollars***				***Footbridge***				***Lifeboat***			
		Pun	NegE	UndE	MAp	Pun	NegE	UndE	MAp	Pun	NegE	UndE	MAp	Pun	NegE	UndE	MAp
***Architect***	Pun	−															
	NegE	.08*	−														
	UndE	-.16***	-.07	−													
	MAp	-.10*	-.10*	.39***	−												
***Smother for dollars***	Pun	.35***	.00	-.06	-.04	−											
	NegE	.07	.41***	-.02	-.03	.18***	−										
	UndE	-.09*	.03	.22***	.14***	-.34***	-.10**	−									
	MAp	-.07	-.01	.13***	.18***	-.41***	-.14***	.54***	−								
***Footbridge***	Pun	.24***	-.01	.00	-.01	.26***	.03	-.03	-.11***	−							
	NegE	.03	.35***	-.03	-.04	.04	.36***	.07	-.06	.17***	−						
	UndE	-.05	.10	.13***	.01	-.08	.05	.16***	.21***	-.35***	-.10**	−					
	MAp	-.03	-.02	.20***	.17***	-.06	.01	.06	.17***	-.28***	-.20***	.42***	−				
***Lifeboat***	Pun	.15***	.00	.04	.04	.16***	.01	-.06	-.10**	.31***	.08	-.12***	-.06	−			
	NegE	.02	.39***	-.00	-.02	.03	.36***	.07	-.02	.06	.44***	.02	-.08	.13***	−		
	UndE	-.02	.08	.12***	.03	.04	.07	.16***	.15***	-.13***	-.02	.30***	.17***	-.26***	-.03	−	
	MAp	-.01	-.03	.18***	.16***	-.07	.02	.09	.15***	-.07	-.06	.16***	.33***	-.22***	-.16***	.49***	−
***Personality***	PF1	-.01	.30***	-.11***	-.12***	.00	.15***	-.04	-.03	-.04	.21***	-.00	-.11***	-.04	.26***	-.00	-.08
	PF2	-.05	-.00	.01	-.00	-.01	.04	.01	-.00	-.02	.07	-.04	-.01	-.00	.04	-.05	-.05
	PF3	.13***	-.07	.31***	.23***	.00	-.08*	.11***	.07	.18***	-.06	.00	.23***	.24***	-.06	.00	.18***
	PF4	-.02	.14***	-.07	-.05	-.01	.12***	-.02	-.01	-.03	.15***	-.00	-.04	-.03	.14***	.01	-.02
	PF5	.02	.01	-.03	.10*	-.07	.01	.06	.09	.18***	.19***	-.24***	-.00	.11***	.14***	-.06	-.01

Kendall’s τ rank correlations,

* *p* < 2.5e-4,

** *p* < 5.0e-5,

*** *p* < 5.0e-6, two-tailed, Bonferroni-corrected. Abbreviations: Punishment (Pun), Negative emotions (NegE), Understanding emotions (UndE), Moral Appropriateness (MAp).

### 3.3 Motive for killing ⨯ avoidability of victims’ death

**Fig 1:**
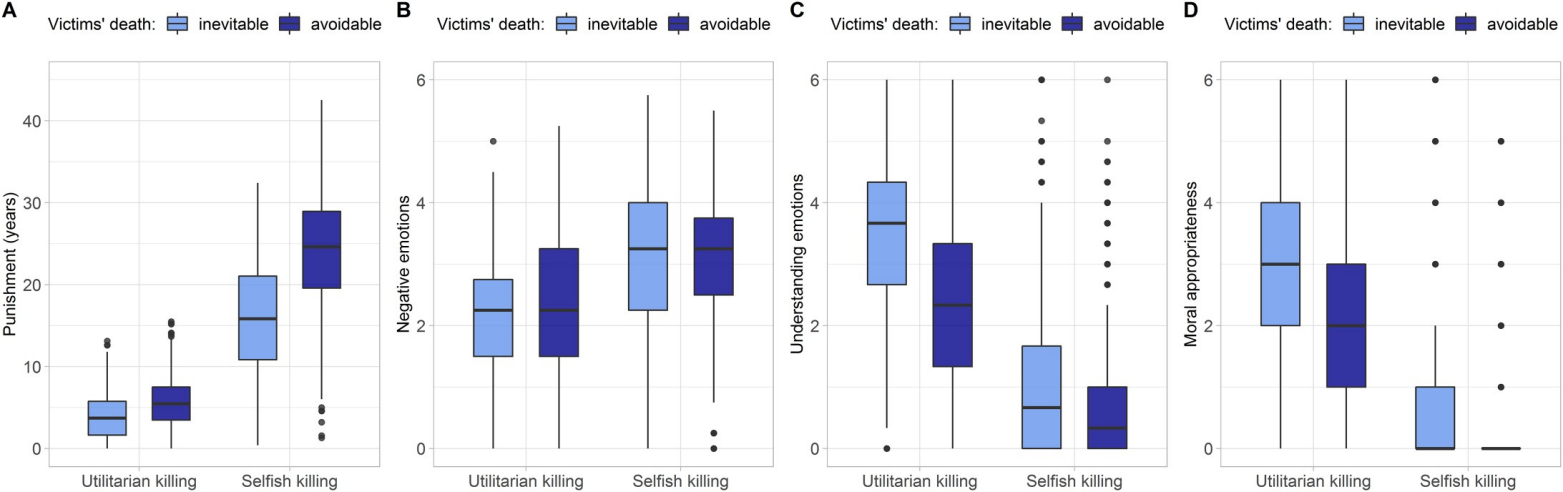
Influence of motive for killing and avoidability of victims’ death. On (A) assigned punishments, (B) observers’ negative and (C) understanding emotions towards perpetrators, and (D) moral appropriateness ratings.

Table 4 summarizes the results of the linear mixed effect models testing the main and interaction effects of motive for killing ⨯ avoidability of victims’ death ⨯ personality. Independent of personality differences, the two content factors (motive for killing, avoidability of victims’ death) had large-sized main effects on all outcome variables. Furthermore, their interaction had an effect on assigned punishment, understanding emotions, and perceived moral appropriateness (Fig 1).

**Table 4:** Results of linear mixed effect models analyzing the interaction of motive for killing ⨯ avoidability of victims’ death ⨯ personality (*N* = 1004).

	Imprisonment duration (years)						Negative Emotions [0,6]						Understanding Emotions [0,6]						Perceived moral appropriateness [0,6]					
Model factors	*b*	*SE*	β	*t*	*p*	η^2^_p_	*b*	*SE*	β	*t*	*p*	η^2^_p_	*b*	*SE*	β	*t*	*p*	η^2^_p_	*b*	*SE*	β	*t*	*p*	η^2^_p_
Intercept	**3.94**	**0.17**		**22.88**	**< .001**		**2.22**	**0.03**		**72.41**	**< .001**		**3.47**	**0.04**		**95.35**	**< .001**		**2.85**	**0.03**		**82.31**	**< .001**	
Motive for killing	**11.85**	**0.21**	**.59**	**57.69**	**< .001**	**.77**	**0.76**	**0.03**	**.34**	**23.61**	**< .001**	**.25**	**-2.47**	**0.04**	**-.76**	**-55.08**	**< .001**	**.55**	**-2.22**	**0.04**	**-.72**	**-49.98**	**< .001**	**.51**
Avoidability	**1.64**	**0.21**	**.08**	**7.98**	**< .001**	**.28**	**0.15**	**0.03**	**.07**	**4.76**	**< .001**	**.01**	**-1.08**	**0.04**	**-.33**	**-24.06**	**< .001**	**.11**	**-1.07**	**0.04**	**-.35**	**-24.14**	**< .001**	**.14**
PF1	0.09	0.20	.01	0.46	.648	.00	**0.34**	**0.04**	**.30**	**9.39**	**< .001**	**.04**	0.01	0.04	.01	0.24	.812	.00	**-0.20**	**0.04**	**-.13**	**-5.08**	**< .001**	**.01**
PF2	0.03	0.18	.00	0.14	.889	.00	0.06	0.03	.05	1.89	.059	.00	-0.07	0.04	-.04	-1.89	.058	.00	-0.06	0.04	-.04	-1.59	.113	.00
PF3	**0.77**	**0.19**	**.08**	**4.05**	**< .001**	**.01**	0.03	0.03	.02	0.78	.434	.00	0.02	0.04	.01	0.38	.702	.01	**0.32**	**0.04**	**.21**	**8.36**	**< .001**	**.03**
PF4	0.02	0.19	.00	0.10	.923	.00	0.02	0.03	.02	0.53	.598	.00	0.04	0.04	.02	0.96	.336	.00	0.05	0.04	.03	1.18	.240	.00
PF5	0.23	0.18	.02	1.29	.196	.00	**0.11**	**0.03**	**.10**	**3.59**	**< .001**	**.00**	**-0.27**	**0.04**	**-.16**	**-7.13**	**< .001**	**.02**	-0.05	0.04	-.03	-1.32	.186	.00
Motive for killing x avoidability	**7.24**	**0.29**	**.31**	**24.91**	**< .001**	**.16**	-0.02	0.05	-.01	-0.34	.737	.00	**0.84**	**0.06**	**.22**	**13.31**	**< .001**	**.05**	**0.63**	**0.06**	**.18**	**10.09**	**< .001**	**.03**
Motive for killing ⨯ PF1	-0.16	0.24	-.01	-0.68	.498	.00	**-0.12**	**0.04**	**-.07**	**-3.10**	**.002**	**.00**	0.03	0.05	.01	0.62	.534	.00	**0.19**	**0.05**	**.09**	**3.67**	**< .001**	**.01**
Avoidability ⨯ PF1	-0.04	0.24	.00	-0.16	.872	.00	-0.02	0.04	-.01	-0.63	.529	.01	0.01	0.05	.01	0.23	.818	.00	-0.04	0.05	-.02	-0.75	.451	.00
Motive for killing ⨯ PF2	0.00	0.22	.00	0.01	.992	.00	0.01	0.03	.00	0.23	.819	.00	0.10	0.05	.04	2.08	.038	.00	0.02	0.05	.01	0.49	.622	.00
Avoidability ⨯ PF2	-0.11	0.22	-.01	-0.48	.628	.00	0.02	0.03	.02	0.73	.468	.00	0.04	0.05	.02	0.94	.348	.00	0.05	0.05	.02	1.16	.244	.00
Motive for killing ⨯ PF3	**-0.66**	**0.23**	**-.05**	**-2.91**	**.004**	**.00**	**-0.11**	**0.04**	**-.07**	**-3.02**	**.003**	**.00**	**0.17**	**0.05**	**.08**	**3.53**	**< .001**	**.02**	**-0.20**	**0.05**	**-.09**	**-4.12**	**< .001**	**.01**
Avoidability ⨯ PF3	-0.03	0.23	.00	-0.14	.891	.00	-0.04	0.04	-.02	-1.00	.316	.00	0.05	0.05	.02	0.98	.328	.01	0.09	0.05	.04	1.85	.064	.00
Motive for killing ⨯ PF4	-0.07	0.23	.00	-0.28	.777	.00	0.08	0.04	.05	2.09	.036	.00	-0.06	0.05	-.02	-1.10	.272	.00	-0.08	0.05	-.03	-1.53	.127	.00
Avoidability ⨯ PF4	-0.03	0.23	.00	-0.15	.883	.00	0.05	0.04	.03	1.31	.191	.00	0.00	0.05	.00	0.03	.980	.00	-0.02	0.05	-.01	-0.48	.628	.00
Motive for killing ⨯ PF5	**-0.69**	**0.21**	**-.05**	**-3.26**	**.001**	**.01**	**-0.10**	**0.03**	**-.07**	**-3.15**	**.002**	**.02**	**0.36**	**0.05**	**.15**	**7.77**	**< .001**	**.05**	**0.19**	**0.05**	**.09**	**4.26**	**< .001**	**.01**
Avoidability ⨯ PF5	0.49	0.21	.03	2.33	.020	.00	**0.12**	**0.03**	**.07**	**3.54**	**< .001**	**.00**	**-0.29**	**0.05**	**-.12**	**-6.24**	**< .001**	**.01**	0.00	0.05	.00	0.07	.945	.00
Motive for killing ⨯ avoidability x PF1	0.16	0.34	.01	0.48	.632	.00	**0.28**	**0.05**	**.12**	**5.30**	**< .001**	**.01**	-0.03	0.07	-.01	-0.38	.705	.00	0.03	0.07	.01	0.38	.705	.00
Motive for killing ⨯ avoidability x PF2	-0.50	0.31	-.02	-1.62	.105	.00	-0.05	0.05	-.02	-1.05	.296	.00	-0.02	0.07	-.01	-0.31	.759	.00	-0.02	0.07	-.01	-0.25	.799	.00
Motive for killing ⨯ avoidability x PF3	**0.96**	**0.32**	**.05**	**3.01**	**.003**	**.00**	**0.16**	**0.05**	**.07**	**3.27**	**.001**	**.00**	**0.22**	**0.07**	**.07**	**3.14**	**.002**	**.00**	0.00	0.07	.00	-0.05	.958	.00
Motive for killing ⨯ avoidability x PF4	0.09	0.33	.00	0.29	.774	.00	-0.10	0.05	-.04	-1.89	.059	.00	0.03	0.07	.01	0.36	.721	.00	0.06	0.07	.02	0.89	.371	.00
Motive for killing ⨯ avoidability x PF5	0.06	0.30	.00	0.21	.837	.00	**-0.12**	**0.05**	**-.05**	**-2.63**	**.009**	**.00**	0.14	0.07	.04	2.17	.030	.00	-0.11	0.06	-.04	-1.71	.088	.00
Overall model statistic	*F*(23, 3690.89) = 581.43, *p* < 2.2e-16***, *R*^2^ = .790						*F*(23, 3690.89) = 66.20, *p* < 2.2e-16***, *R*^2^ = .590						*F*(23, 3690.89) = 228.82, *p* < 2.2e-16***, *R*^2^ = .624						*F*(23, 3690.89) = 205.23, *p* < 2.2e-16***, *R*^2^ = .590					

Bold-faced values indicate significant effects at *p*_adj_ < 0.0125.

Selfish killings were punished more severely. Prison sentences assigned by observers were on average 11.85 years longer in cases of selfish killings compared to utilitarian ones (Fig 1A). Participants also felt stronger negative emotions (Fig 1B) and were less understanding of selfish compared to utilitarian killings (Fig 1C). Furthermore, they considered selfish killings less morally appropriate than utilitarian killings (Fig 1D). Compared to killings of inevitably dying victims, observers punished the killing of avoidable victims more harshly. Assigned prison sentences were on average 1.64 years longer when the victim’s death could have been avoided (Fig 1A). Observers also reported stronger negative emotions (Fig 1B) and less understanding sentiments towards killers of avoidable victims (Fig 1C) and rated their murder as less morally appropriate (Fig 1D). Furthermore, interaction effects (motive for killing ⨯ avoidability of victims’ death) indicated that the selfish killing of avoidable victims (i.e., the *architect* scenario) elicited particularly severe punishments (Fig 1A), reduced understanding emotions (Fig 1C) and decreased moral appropriateness ratings (Fig 1D).

### 3.4 Main and interaction effects of observer personality

**Fig 2:**
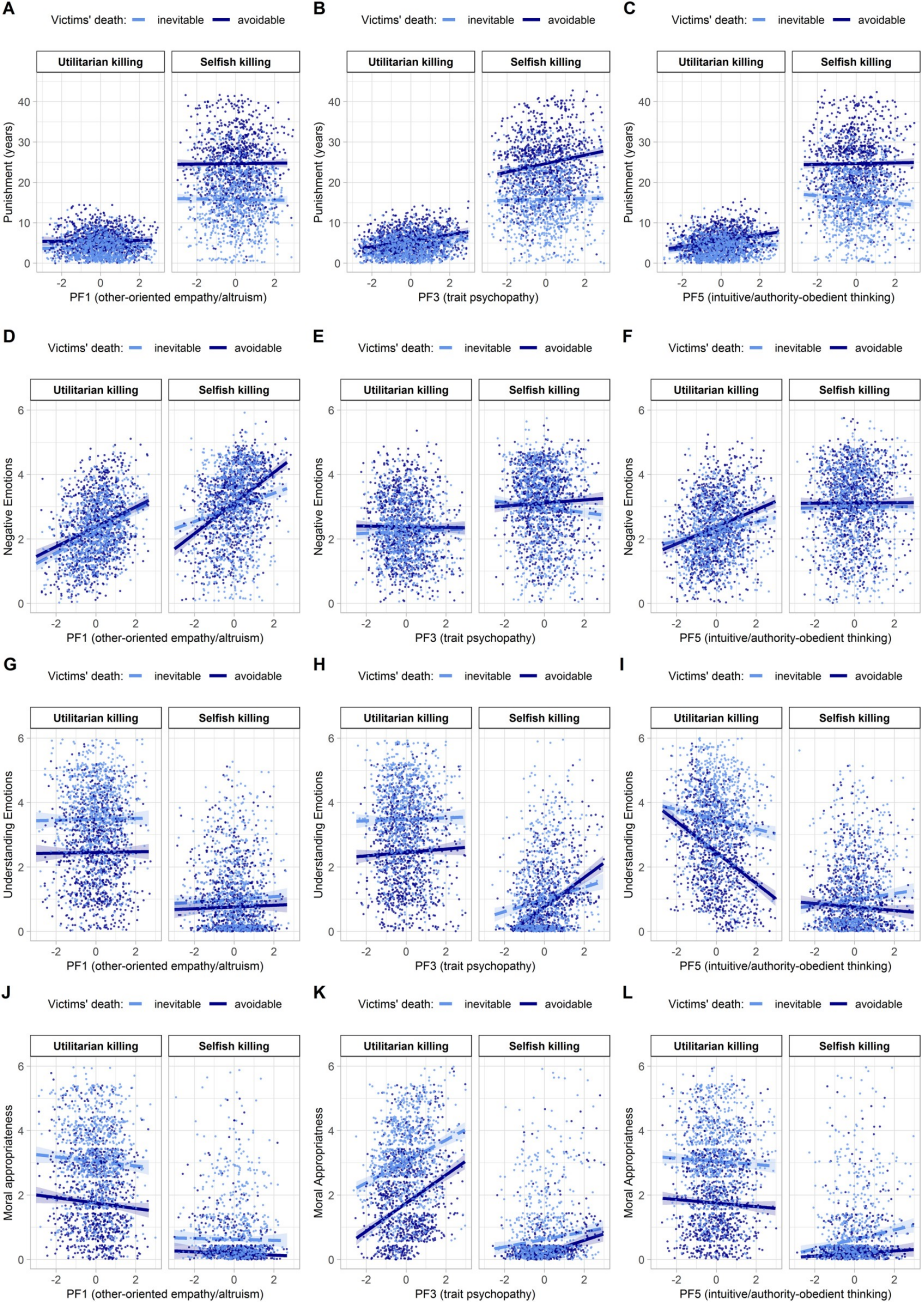
Influence of observer personality factors. On (A–C) the assigned punishments, the observers’ (D–F) negative emotions and (G–I) understanding emotions towards perpetrators, and (J–L) the moral appropriateness rated by observers.

The personality factors **PF1** (other-oriented empathy, altruism), **PF3** (trait psychopathy), and **PF5** (intuitive/authority-obedient thinking, victim justice sensitivity, low NFC) showed main and interaction effects, whereas *no* effects of PF2 (anxiety, personal distress, low self-esteem) and PF4 (other-oriented justice sensitivity) were observed (Table 4). The interplay of the content factors with PF1, PF3, and PF5 is displayed in Fig 2. Additional results of all post-hoc linear contrast tests can be found in the Supplement.

In detail, participants with more marked **PF1** characteristics (other-oriented empathy, altruism) reported more intense negative emotions towards perpetrators across all conditions. Towards selfish killings, the increase of negative emotions was steeper in case of avoidable victims and weaker in case of inevitable victims (post-hoc linear contrast, Δ*b*(*SE*) = 0.26 (0.04), *p*_Holm_ < .00025***) (Fig 2D). Moreover, they judged all instrumental killings as less morally appropriate (Fig 2J).

**PF3** characteristics (trait psychopathy) were linked to harsher punishments across nearly all conditions. The only exception was punishment for selfish killing of inevitable victims (i.e., *smother for dollars*) which did not increase with higher PF3 scores (Δ*b*(*SE*) = 0.74 (0.18), *p*_Holm_ = .00037**) (Fig 2B). Observers with higher PF3 scores reported slightly less negative emotions towards the selfish killing of inevitable victims and slightly more negative emotions towards the selfish killing of avoidable victims (Δ*b*(*SE*) = 0.13 (0.04), *p*_Holm_ = .00132**) (Fig 2E). Higher PF3 scores were also linked to a specific increase in understanding emotions for *selfish* killings (Δ*b*(*SE*) = 0.29 (0.04), *p*_Holm_ < .00025***), with the increase in understanding emotions being stronger for the selfish killing of avoidable compared to inevitable victims (Δ*b*(*SE*) = 0.27 (0.05), *p*_Holm_ < .00025***) (Fig 2H). At the same time, observers with higher PF3 scores perceived all killings, but particularly utilitarian killings, as more morally appropriate (Δ*b*(*SE*) = 0.22 (0.04), *p*_Holm_ < .00025***) (Fig 2K).

Higher **PF5** scores (intuitive/authority-obedient thinking and low NFC) were linked to increased punishment severity for utilitarian killings (Δ*b*(*SE*) = 0.66 (0.15), *p*_Holm_ < .00025***) as well as harsher punishments for the killing of avoidable victims (Δ*b*(*SE*) = 0.52 (0.15), *p*_Holm_ = .00139**) (Fig 2C). Higher PF5 scores were also associated with increased negative emotions specifically towards utilitarian killings (Δ*b*(*SE*) = 0.20 (0.02), *p*_Holm_ < .00025***). Observers with high PF5 scores differentiated less between the motives for killing (Fig 2F). Moreover, negative emotions towards the utilitarian killing of avoidable victims increased stronger with PF5 compared to inevitable victims (Δ*b*(*SE*) = 0.12 (0.03), *p*_Holm_ = .00132**).

Conversely, observers with lower PF5 scores (deliberative/independent thinking, high NFC) showed milder punitive and negative emotional responses to utilitarian killings and differentiated less between avoidable and inevitable victims (Fig 2C and 2F). Also, observers with lower PF5 scores reported more understanding emotions for utilitarian killings and differentiated less between avoidable and inevitable victims (Fig 2I). Conversely, higher PF5 scores were linked to a specific decrease of understanding emotions towards utilitarian killings (Δ*b*(*SE*) = -0.33 (0.03), *p*_Holm_ < .00025***) and the killing of avoidable victims (Δ*b*(*SE*) = -0.24 (0.03), *p*_Holm_ < .00025***). Moreover, PF5 was linked to a strong reduction of understanding emotions towards the utilitarian killing of avoidable as compared to inevitable victims (Δ*b*(*SE*) = -0.33 (0.05), *p*_Holm_ < .00025**) (Fig 2I). Higher PF5 scores were linked to decreased moral appropriateness ratings for utilitarian killings. However, they were also associated with increased moral appropriateness ratings for selfish killings (Δ*b*(*SE*) = 0.15 (0.03), *p*_Holm_ < .00025***) (Fig 2L).

### 3.5 Results of supplementary analyses

In line with the main analysis, the supplementary scenario contrasts indicated significant effects of PF1, PF3, and PF5, whereas no effects were observed for PF2 and PF4. Detailed results are listed in the supplement.

## 4. Discussion

This study explored whether personality characteristics account for inter-individual differences in third parties’ judgment and punishment of instrumental killings in moral dilemmas. We investigated individual differences in the consideration of (i) utilitarian motives to kill and (ii) the avoidability of the victims’ death as mitigating factors. To this end, we compared self-interested straight-up murder of an avoidable victim (*architect*) as well as an inevitably dying person (*smother for dollars*) with utilitarian killings of an avoidable victim (*footbridge*) and inevitably dying persons (*lifeboat*).

In general, observers took both the utilitarian motive and the avoidability of victims’ death as mitigating factors into account and assigned milder punishments. Correspondingly, observers considered utilitarian killings as morally more appropriate and perceived less negative and more understanding emotions towards the respective perpetrators. In line with previous findings [27], observers considered killings more morally appropriate when the victims’ deaths were inevitable. Correspondingly, they felt less negative and more understanding emotions for the killing of inevitable victims and assigned less harsh punishments. Additionally, we observed an interaction of the two factors. Observers judged the selfish killing of avoidable victims as particularly morally inappropriate, reported markedly low understanding emotions towards the perpetrator, and assigned the harshest punishments. However, this overall response pattern varied substantially in association with the observers’ personality. Below we will discuss our most important findings in close relationship to previous studies.

### 4.1 Other-oriented empathy and altruism

*Empathy* and *altruism* (**PF1**) were associated with differences in negative emotions and perceived appropriateness of instrumental killings. Highly empathic/altruistic observers reported more intense negative emotions towards the perpetrators in *all* scenario conditions, but particularly in response to the selfish killing of avoidable victims. Consistently, empathic/altruistic observers judged *all* instrumental killings as morally less appropriate. In line with these findings, previous studies showed that more empathic individuals report more emotional discomfort during moral decision making from the first-party perspective as they get more immersed in the fate of threatened individuals and imagine harmful acts more vividly [44,46]. As a result, more empathic individuals have been found to favor deontic choices in order to prevent instrumental harm [28,47–51].

Importantly, in our study, empathy/altruism was neither associated with differences in punishment severity nor understanding emotions towards perpetrators. High empathic/altruistic observers indicated no elevated pity or compassion with the perpetrators in *footbridge* or *lifeboat*, although these protagonists were in desperate situations. However, our supplementary analyses of the scenarios *crying baby* and *Sophie’s choice* revealed that empathic/altruistic observers exhibited more negative *and* more understanding emotions towards mothers who had to make the desperate decision to sacrifice their child to protect other family members (see S1 File). Thus, third parties’ empathic tendencies may not be limited to the fate of the victims but could also lead to an increased understanding for a protagonists’ desperate situation. Future studies need to investigate whether emotional empathy for perpetrators is limited to dilemma situations in which the fate of protagonists’ own relatives is at stake.

### 4.2 Empathic distress and trait anxiety

We found no associations between *empathic distress* and trait *anxiety* (**PF2**) with differences in third-party responses to instrumental killings. Individuals with high scores in PF2 traits are prone to feel fear, discomfort or shame when confronted with others’ suffering and report low self-esteem [56,57]. To end this clearly aversive state, they have been found to be quick to help—but even more frequently, they tended to elude respective situations, which ultimately results in less helping behavior [31]. Congruently, we found a negative association between empathic distress/anxiety and other-oriented empathy/altruism. Previous studies also reported a link between less utilitarian moral choices (in from the first-party perspective) and empathic distress as well as, albeit inconsistently, anxiety [55,59,60].

It has been speculated that highly empathically distressed/anxious individuals might quickly choose deontic solutions in order to end their own negative feelings caused by imagining the suffering of others [55,56]. However, in our study, trait empathic distress/anxiety was not associated with stronger negative emotions in any scenario condition. Moreover, trait empathic distress/anxiety did not account for differences in observers’ moral judgments or punishment behavior. Correspondingly, previous research found trait anxiety to be associated with self-reported reactive aggression [59], but found no link between trait anxiety and third-party punishments in crime vignettes [58]. Future research is needed to assess self-oriented emotions (i.e., anxiety, discomfort, shame or helplessness) to further elucidate the emotional state of highly empathic distressed/anxious individuals when judging criminal acts.

### 4.3 Trait psychopathy

Trait *psychopathy* (**PF3**) was associated with punishment severity, moral appropriateness ratings and understanding emotions. Third-party observers with higher trait psychopathy judged *all* instrumental killings as more morally appropriate, particularly when perpetrators had utilitarian motives. However, they also reported more understanding of selfish killings, particularly the selfish killing of avoidable victims (*architect* scenario). Thus, individuals with higher trait psychopathy did not selectively excuse utilitarian killings, but showed increasing understanding emotions for killing in general and in particular for killing of avoidable victims.

These findings complement previous studies using a first-party perspective: Trait psychopathy was associated with a weaker inclination to deontic decisions, but *not* with an increased tendency towards utilitarian options [51,72]. Individuals with stronger psychopathic tendencies care less about deontic moral norms, especially fairness and harm avoidance [66–68], and seem less sensitive for the consequences of their decisions. Thus, they are generally less reluctant to make decisions and take action [73]. Their disrespect for deontic rules has been found to be accompanied by reduced aversive emotional responses to witnessing or inflicting harm [63], which might be due to reduced empathy for victims or individuals in need [49,51,66,67,74–76]. In turn, low empathic concern for others is linked to less anger in observers witnessing unfair treatment of others [30]. In our study, however, higher trait psychopathy was not linked to decreased negative emotions towards perpetrators in general. Instead, there was a specific reduction of negative emotions towards selfish killers of inevitable victims as well as increased negative emotions towards selfish killers of avoidable victims. Furthermore, no effect of trait psychopathy was found on negative emotions towards utilitarian killings.

Observers with higher trait psychopathy (and low perpetrator injustice sensitivity) imposed in our study harsher punishments across all conditions with the exception of the selfish killing of inevitable victims (*smother for dollars*). One possible explanation for this tendency of harsher punishment might be their decreased aversion to perform harmful acts [63] and reduced empathy [75,76], albeit here towards the perpetrator. However, observers with higher psychopathy scores also regarded these killings as *less* morally reprehensible and reported more understanding emotions. Similar disparities between moral judgment and punitive reaction were already reported in previous studies on trait psychopathy. Observers with higher trait psychopathy appear to punish less out of retaliation or because an offender deserves punishment [70]. Thus, punishment in these cases seems less motivated by reactive aggression [59] or moral emotions (e.g., anger or indignation). Instead, it has been suggested that individuals with higher trait psychopathy punish those perpetrators harsher that are likely to reoffend [77]. Thus, punishment might predominantly serve as deterrence of future transgressions and represent a “cold” proactive aggression [59,77]. Similar findings come from research on economic games. Individuals with higher psychopathy scores invest more effort to sanction players who try to take unfair advantages while violating rules to gain advantages themselves [78,79].

In sum, our findings are in line with the notion that trait *psychopathy* is associated with reduced aversion to instrumental harm. Individuals with higher self-reported psychopathy considered killings less morally reprehensible but nevertheless imposed harsher punishments. However, it should be kept in mind that our study did not investigate clinically psychopathic individuals but trait psychopathy in a community sample. Additional experiments are needed to further elucidate the roles of trait psychopathy as well as “cold” proactive aggression vs. retaliation on third-party judgment and punishment. Future studies should comprise community as well as clinical samples.

### 4.4 Other-oriented justice sensitivity

No effects of other-oriented *justice sensitivity* (**PF4**) on responses to instrumental killings emerged in our study. Contrariwise, in previous studies, individuals with higher other-oriented justice sensitivity rated immoral behavior in moral dilemmas as less permissible and showed more empathic concern and perspective taking as well as lower trait psychopathy [62]. They also responded with more anger and harsher punitive reactions to observed unfairness in economic games [81]. The inconsistency between our findings and the literature might be due to differences in the respective violated moral domains examined here compared to others studies (i.e., physical harm vs. unfair distribution of money). It might also stem from the fact that injustice in economic games can be reversed, i.e., victims of unfair distributions can be reimbursed while murder victims cannot be revived.

### 4.5 Need for cognition and intuitively/authority-obediently thinking

The personality traits comprising **PF5** were associated with differences in punitive, emotional and cognitive responses to instrumental killings. Intuitively/authority-obediently thinking observers reported more negative and less understanding emotions for utilitarian killings than observers with high NFC scores. Moreover, their emotional reactions differed less between utilitarian killings and selfish murder, specifically in case of the utilitarian killing of avoidable victims. Consistently, they punished utilitarian killings more harshly, especially the utilitarian killing of avoidable victims. In contrast, deliberatively/authority-independently thinking observers reported less negative and more comprehensive emotions towards utilitarian perpetrators and differentiated less between the avoidable and inevitable victims in case of utilitarian and selfish killing. They also punished utilitarian killings of avoidable vs. inevitably dying victims similarly and generally less severe than highly intuitively/authority-obediently thinking observers did.

Our findings correspond with moral decision patterns in the first-party perspective. Participants with greater *faith in intuition* made more deontic and less utilitarian judgments [91,118], whereas participants prone to deliberate reasoning made more utilitarian judgments [28,84,119]. Deliberate thinking styles (e.g., higher NFC) are associated with a greater concern for optimizing overall welfare, but *not* with reduced concern for the deontic principle of preventing instrumental harm [88]. It has been suggested that individual differences in cognitive motivation and/or ability lead to differences in encoding and analyzing moral issues and related contextual information. Indeed, studies have demonstrated that utilitarian judgment tendencies are selectively impaired by cognitive load, while deontic tendencies remained unchanged [28,93]. Sargent [92] theorized that high-NFC individuals are more *able* and/or *willing* to mobilize cognitive efforts for deliberating perpetrators’ motives, reasons, and situational constraints when judging behavior. In turn, they rely less on situationally invariant deontic judgment templates and therefore, are less willing to support harsh punitive responses to crime. Conversely, individuals with authority-obedient mindsets were found to be less likely to examine or integrate more than one perspective on a (moral) problem, especially concerning questions on existential issues such as life and death [94]. Rather they “solved” moral problems through a quick and situationally invariant application of deontic moral rules [11,28,94–96]. Previous studies also concluded that authority-obedient individuals tend to experience pronounced aggressive feelings and to impose harsher sanctions on violators of deontic norms in general [97].

However, our own results revealed a more complex picture: Compared to high-NFC observers, intuitively/authority-obediently thinking observers did neither generally punish selfish murder more harshly, nor did they exhibit more negative emotions towards selfish killers. Thus, intuitive/authority-obedient thinking did not result in more severe retaliation for deontic rule transgressions (here: instrumental killing) in general. They also assigned milder punishments to perpetrators with utilitarian motives, although to a lesser degree than high-NFC observers. Moreover, they reported more understanding and less negative emotions in reaction to utilitarian killings, particularly in the case of inevitable harm to victims. Thus, intuitively/authority-obediently thinking observers were not insensitive to situational differences. However, they attached greater importance to the avoidability of harm to individual victims than to minimizing net harm. Conversely, more deliberatively/authority-independently thinking observers saw utilitarian (as opposed to selfish) motives as a strong mitigating factor while the (in-)evitability of harm was less important for their judgement.

### 4.6 Limitations and future research

Although the current study involved assessment of emotional, cognitive, and punitive responses and presents a comprehensive investigation on which levels morality-associated personality traits are associated with third parties’ judgment of homicides, and had a large sample size which allowed for detecting even small effects, it also had several limitations and thus should be followed by further research.

Among the general limitations are sample characteristics. The present sample consisted mainly of young, Central/Western Europeans with a background in academics who identified themselves as either atheist or Christian. Sanctioning behavior could differ in populations with different educational, moral, religious, or cultural background. Furthermore, we asked non-judicial participants for their intuitive emotional, cognitive, and punitive reactions. Their decision might not be comparable to the rationally deliberated verdict of a professional judge. However, judging real-life crimes is not always delegated to professional judges. Depending on the legal system, a jury consisting of lay persons might be in charge of deciding whether alleged perpetrators are guilty and how they should be punished. Thus, findings on moral judgements by lay persons have relevance in real-life. Furthermore, there is evidence that German lay persons’ judgments on violent crimes did not considerably differ regardless whether they were instructed to judge intuitively or elaborately, rationally reasoned [120].

Further limitations concern the method of data collection and employed paradigms. Online studies such as ours are characterized by an innately less controllable experimental setting. Also, while moral dilemmas have been widely used in research, their usefulness for predicting moral behavior in real-life situations is debatable. A recent study using a virtual reality implementation of the *footbridge* dilemma showed that a clear majority of all participants would push the large stranger off the bridge [121,122], contradicting the ‘classical’ deontic response pattern found in almost all previous studies using written moral dilemmas. This disparity could indicate an unconscious moral self-deception among participants, which only comes to light when they actually have to have to take action under (more) realistic conditions [123,124]. In sum, additional research is needed on the validity of punitive responses in written moral dilemmas.

It should also be noted that many moral dilemmas used in research are somewhat ambiguous regarding key content features which might result in different interpretations. For instance, the classification of described actions in moral scenarios as “utilitarian” vs. “selfish” might not be as clear-cut in some cases, i.e. the described actions might be perceived as selfish *or* utilitarian *or* both depending on how participants interpret them. For instance, in *lifeboat*, the action of the officer may be seen both utilitarian and selfish. Since we have no ratings from participants on how they themselves interpreted the motive behind the killings, such alternative interpretations cannot be ruled out. Thus, in future studies the scenarios should be systematically rated to ensure correct classification. Specifically, participants could be asked to decide whether an action’s effects are in the best interest of (i) the agent himself, (ii) all affected people, or (iii) both. In addition, they might rate whether, in their opinion, a utilitarian or a selfish motive is the stronger impetus of an agent’s actions. This approach would help to clarify the classification of ambiguous dilemmas and to identify scenarios which might need to be discarded because they are too confounded. Furthermore, some of the dilemmas we used as filler scenarios also shared, although confounded, the content features that were of interest in this study. While we provide additional analyses of them in the supplement, systematic future investigations are warranted to ensure that the effects of content features may be generalized on dilemmas different from the ones employed in this study.

Additional concerns might be raised regarding the use of a within-subject design in this study. We chose this economic approach to investigate inter-individual differences across different hypothetical situations. However, within-subject designs have disadvantages: In our study, we examined two clearly defined features of killing scenarios using four moral dilemmas. However, presenting only these four dilemmas in direct sequence would have made the targeted contrasts very obvious, which could have influenced participants’ responses. In fact, there is evidence for strong carry-over effects [104] when presenting moral dilemmas in sequence. We tried to avoid this by embedding the scenarios in question in a randomly chosen series of scenarios derived from widely used moral dilemmas. A second disadvantage of our within-subject design is the fact that we could not manipulate scenario features within the same overall story, which would have provided more clear-cut stimulus material. However, using highly similar stories can create boredom and monotony among participants, which in turn might result in less attention to the small but pivotal differences between scenarios and eventually lead to unreliable responses. Thus, the use of (additional) heterogeneous scenarios also served to ensure participants’ motivation and compliance. Still, as a result of the heterogeneity between the analyzed dilemmas, it could be argued that the milder punishment of the protagonist in *lifeboat* might not only be due to fact that the passengers’ death was most likely inevitable (compared to the clearly evitable death of the large stranger in *footbridge*). An alternative explanation for the milder punishment would be that that the protagonist in *lifeboat* also acted out of a self-protective motive. Importantly, our supplementary analyses confirmed that the observed personality effects generalize across additional scenarios involving instrumental killings. Thus, these personality traits might influence responses to various content features of moral dilemmas.

Taken together, future studies are necessary to (i) replicate our findings in independent samples with similar as well as different educational, religious, and cultural backgrounds; (ii) examine additional dilemma-content features and personality traits that were beyond the scope of this study; (iii) explore the influences of personality on real-life moral decisions. The advantages and disadvantages of within- and between-subject designs need to be carefully considered.

## 5. Conclusions

Overall, third-party observers regarded utilitarian motives for killing as well as the avoidability of victims’ death as mitigating factors when judging and punishing instrumental killings in moral dilemmas. Punitive reactions were accompanied by judgments of moral inappropriateness as well as higher negative emotions and lower understanding emotions towards perpetrators. The personality factors (i) other-oriented empathy/altruism (PF1), (ii) empathic distress/trait anxiety (PF2), and (iii) other-oriented justice sensitivity (PF4) were not associated with differences in third-party punishment. Conversely, trait psychopathy (PF3) was associated with harsher punishment and simultaneously with (i) a generally lower concern about the violation of deontic principles not to kill, (ii) a higher moral appreciation of a utilitarian motive to kill, and (iii) a reduced concern about the killing of avoidable victims. Observers with a deliberate/authority-independent thinking style (low PF5) considered a utilitarian motive for killing as a strong mitigating factor and were less concerned about the avoidability of harm to the victims. In contrast, observers characterized by intuitive/authority-obedient thinking (high PF5) were particularly concerned about the prevention of avoidable harm, but considered a utilitarian motive for killing as less mitigating when punishing.

In sum, our exploratory study provides evidence that personality differences are associated with third-party judgments and punishments of homicides to a practically relevant magnitude. Our findings may serve as starting point for specific hypotheses in future research. Since personality differences account for behavioral differences even in “strong situations” [125] like homicides, even stronger personality-related differences in altruistic behavior and moral decision making are to be expected in weaker, less clear-cut situations [126]. In various real-life contexts [127], investigating the role of personality might help to explain why people remain silent and others intervene when observing moral misbehavior.

## Supporting information

S1 File (PDF)
