# Supplementary material for: When the killing has been done: Exploring associations of personality with third-party judgment and punishment of homicides in moral dilemma scenarios
Source: PLoS One. 2020 Jun 30;15(6):e0235253. doi: 10.1371/journal.pone.0235253 (PMC7326181; doi:10.1371/journal.pone.0235253)
Supplement: S1 File — (PDF) [file pone.0235253.s001.pdf]

When the killing has been done: Exploring associations of personality with third-party judgment and punishment of homicides in moral dilemma scenarios

# - Supplemental Material -

Alexander Behnke, Anja Strobel, & Diana Armbruster

Stimulus material

1 Moral scenarios

Sixteen dilemma scenarios were presented. Mostly, they were chosen from a number of moral declaims previously used in other studies (e.g., Greene, Sommerville, Nystrom, Darley, & Cohen, 2001). Since participants were to judge the actions of others, all dilemmas were rephrased into a third-party perspective. Also, at the end of each dilemma the course of action chosen by the protagonist was given labeled *outcome*.

To elicit sanctioning behavior in the study's participants, only those options were presented as protagonists' choice which had been judged as the immoral one by the majority of subjects in previous studies (e.g., Cummins & Cummins, 2012). Depending on the severity of immoral behavior in the chosen option as well as on characteristics of the alternative option, the percentage of person rejecting the "immoral" option varies considerably across dilemmas. This finding was confirmed in numerous studies using dilemmas in the first-person perspective. In our study, the percentage of participants willing to judge and sanction the actions of others' immoral choices showed comparable variation. It should be noted, that while English versions of the dilemma are given here, German versions were used in the study.

The following general instruction was displayed in the beginning:

> *Please read the following story carefully. Try to imagine that you are learning about this story from TV, news or the internet.*
>
> *The story summarizes a problem a protagonist is facing and the two alternative courses of action. Following, the action eventually taken by the protagonist is described with its resulting consequences.*

- *Please judge the protagonist's action according to your own thoughts and feelings.*
- *There are no right or wrong answers, only your personal opinion.*
- *Please complete this survey by yourself.*

Hard Times

In a developing country, a persistent drought has devastated the crops of a poor family for the second year in row. Death because of hunger is imminent. The sons, ages eight and ten, are too young to go to work in the city, but the daughter could fare better. A rich businessman looks for young girls for shooting sexually explicit movies. He promises enough money to feed the family for many years.

If nothing is done, the family will starve and several members of the family will die.

The only way for the father to avoid the death of his family is to let his daughter be employed in the child pornography industry for one year.

Outcome: The father decided against the employment of his daughter in child pornography. Due to this decision his daughter did not have to participate in pornographic movies; but several family members starved to death.

Epidemic

A deadly epidemic rages in a poor country. The required drug to treat the disease is scarce and cannot be produced at a faster rate. Genetic differences in blood platelets require one part of the population (x type) to be treated with a dose five times as high as the rest of the population needs (y type).

If nothing is done the epidemic will spread and kill millions.

The only way for the head of disaster management to rescue as many people as possible is to refuse treating the x-type population. Thereby, five persons with the y-type can be treated with the drug for each x-type individual.

Outcome: The head of disaster management decided against refusing to treat x-type individuals. Due to this decision, numerous x-type individuals were rescued as well but for each of them five y-type individuals died due to medication shortage.

Crying Baby

Enemy soldiers take over a village. They have orders to kill all black civilians. A white woman, her black husband, and their black baby have sought refuge together with a number of other black people in a remote cellar. The baby begins to cry loudly.

If nothing is done the crying will summon the soldiers who will kill all black people hidden in the cellar; including the white woman's baby and husband.

The only way to avoid detection is for the woman to cover the mouth of the baby to block the sound; however, the baby will be smothered.

Outcome: The white woman decided to smother her baby. Due to this decision the people in the cellar remained undetected and survived; except for the baby who died.

Standard Fumes

Deadly fumes caused by a cable fire are rising through an intensive care unit's ventilation system. In one room of the unit are five non-transportable patients while in another room is a single non-transportable patient.

If nothing is done the fumes will rise up into the room shared by the five patients and kill them.

The only way for the fire department's officer-in-charge to avoid the deaths of these patients is to switch the ventilation control, which will cause the fumes to bypass into the room of the single patient who will be killed.

Outcome: The officer-in-charge decided against switching the ventilation control. Due to this decision the single patient survived; but five patients were killed.

Hostages

Two members of an aid organization were taken hostage by a terror organization. To extort money for new weapons from the hostages' country of origin the terror organization demands a ransom of several hundred millions of dollars.

If nothing is done the hostages will be executed by their kidnappers.

The only way for the president of the hostages' country to avoid their deaths is to pay the ransom. Thereby, the terror organization will buy new weapons and become even more dangerous.

Outcome: The president decided against paying the ransom. Due to this decision the terror organization could not acquire more weapons; but the two hostages were killed.

Trolley

A runaway trolley with broken breaks is quickly approaching a fork in the tracks. On the tracks extending to the right is a group of five railway workmen. On the tracks extending to the left is a single railway workman.

If nothing is done the trolley will proceed to the right causing the deaths of the five workmen.

The only way for an engineer overseeing the tracks to avoid the deaths of these workmen is to turn a switch that will cause the trolley to proceed to the left track, causing the death of the single workman.

Outcome: The engineer decided against turning the switch. Due to this decision the single workman survived; but the five workmen were killed.

Speedboat

While a fisherman is fishing from a seaside dock on a remote Caribbean island, he observes a group of tourists boarding a small boat and setting sail for a nearby island. Soon after the group's departure he hears over the radio that there is a violent storm brewing.

If nothing is done the storm will surely intercept the tourists' boat and sink it.

The only way for the fisherman to reach them in time and return them to the island is to follow them with a nearby anchoring speedboat. Since the harbor guard has already finished for the day, the fisherman would have to break open and hot-wire the boat. Thus, considerable damage would result for the boat's owner.

Outcome: The fisherman decided against breaking open and hot-wiring the speedboat. Due to this decision no damage resulted for the boat owner; but the tourists were caught by the storm and drowned.

Sophie's Choice

During wartime a village is occupied. Enemy soldiers face a mother of two young children with the choice which of her children to hand over. At the enemy's headquarters, a doctor performs painful experiments on children that inevitably lead to death.

If no decision is made both children are brought to the doctor.

The only way for the mother to avoid the death of both of her children is to hand over one of them to the doctor, which will result in the death of this child but will save the other.

Outcome: The mother decided to hand over one of her children to the doctor. Due to this decision her second child survived; but her other child died during the doctor's experiments.

Vaccine Policy

A deadly disease spreads. There is a vaccine. As demonstrated by a large body of studies, the vast majority of vaccinated people develop immunity to the disease. However, a very small number of vaccinated people will get a deadly allergic shock due to intolerance of one of the substances in the vaccine.

If nothing is done the deadly disease will spread and kill several millions of people.

The only way for the surgeon general to avoid the deaths of millions is to impose a compulsory vaccination to immunize the population which will, however, result in the death of a small number of people due to severe allergic responses to the vaccine.

Outcome: The surgeon general decided against imposing the compulsory vaccination. Due to this decision millions died of the disease; but none died due to an allergic shock.

Preventing the Spread

To curtail the spread of a deadly and highly contagious disease in Africa quarantine stations are set up. An infected patient is determined to leave the station to die surrounded by his family instead of dying anonymously while quarantined.

If nothing is done the man will leave the station and will spread the disease by infecting many people on the way to his family.

The only way for his physician to stop the man from leaving the quarantine station is to put him against his will in a coma, in which the man will die anonymously.

Outcome: The physician decided against putting the man in a coma. Due to this decision the man could die in the bosom of his family; but the disease spread and many people died.

Modified Lifeboat

A cruise ship has to be abandoned in the Arctic Sea due to an emergency aboard. Lifeboats carry many more people than they are designed to do. They are dangerously low in the water. Since the sea starts to get rough the boats begin to fill with water.

If nothing is done the boats will be filled with water and sink resulting in the drowning of most passengers before the coast guard has arrived.

The only way for the captain to stop the boats from sinking is to throw several passengers overboard, which will cause the boats to lay higher in the water but will also result in the death of these passengers.

Outcome: The captain decided to throw several passengers overboard. Due to this decision the boats did not sink and most passengers were rescued by the coast guard; but all passengers thrown overboard were killed.

Footbridge

A runaway trolley with malfunctioning breaks is heading down the tracks towards a group of five workmen. A pedestrian observes this from a footbridge.

If nothing is done the trolley will overrun and kill the five workmen.

The only way for the pedestrian to avoid the deaths of the five workmen is to push a large stranger who is standing next to him off the bridge onto the tracks below where his large body will stop the trolley but which will also kill the stranger.

Outcome: The pedestrian decided to push the stranger off the bridge. Due to this decision the trolley was stopped and the five workmen were saved; but the stranger was killed.

Country Road

During a Sunday drive along a remote country road, the driver of an expensive convertible discovers a man covered in blood in the roadside bushes. Unfortunately, the weak mobile reception makes it impossible to call for emergency medical services.

If nothing is done the injured man will soon die due to the loss of blood.

The only way for the convertible driver to rescue the injured man is to give him a lift to the next hospital. However, by giving the man a lift, his blood will ruin the leather upholstery of the convertible.

Outcome: The driver decided against giving the injured man a lift. Due to this decision the leather interior of the car was not ruined; but the injured man died.

Smother for dollars

In a hospital lounge a visitor is approached by a young man. The latter explains that his father is incurably ill and holds a substantial life insurance policy that expires at midnight. Since he is in urgent need of the money, he offers the visitor half a million dollars to go up to his father's room and smother his father with a pillow.

If nothing is done the father will definitely die in a few days, but the life insurance policy will have expired.

The only way for the visitor to get the insurance money for the young man and himself is to smother the father today.

Outcome: The visitor decided smothering the father with a pillow. Due to this decision the young man and he received a lot of money from the insurance; but the father was killed.

Transplant

Five patients are treated in a hospital. Each of whom is in critical condition due to organ failing. A healthy man consults the head physician for routine checkup.

If nothing is done the five patients will die due to a shortage of available transplants.

The only way for the head physician to save the lives of the first five patients is to kill the healthy man (against his will) and to transplant his organs into the bodies of the other five patients.

Outcome: The head physician decided to kill the healthy man and to transplant the organs. Due to this decision five patients were saved; but the healthy man was killed.

Architect

A young architect visits one of his construction sites with his boss. His boss is a despicable individual who makes work unendurable for his staff including the architect.

If nothing is done the young architect will go on suffering because of his boss.

The only way for the architect to end this suffering is to push his boss off the building while unobserved and make everyone think that it was a tragic accident.

Outcome: The architect decided to push his boss off the building. Due to this decision the architect did no longer suffer at work; but his boss was killed.

2 Assessment of participants' responses

After reading each dilemma, participants rated their reactions on the subsequent scales. These include the Self-Assessment Manikin scales (Bradley & Lang, 1994), moral emotions ratings (Rudolph & Tscharaktschiew, 2014) as well as additional cognitive ratings. Furthermore, participants decided whether the dilemma's protagonist should be punished for their actions and if so, for how many months or years they should be imprisoned. Preliminary explanations (in squared brackets) were presented only in the first trial.

2.1 Self-Assessment Manikin Scales

[Please indicate how you have felt after reading the story. Try to answer **intuitively**. Please choose those figure, which best represents your current state of mind.]

How do you feel right now?

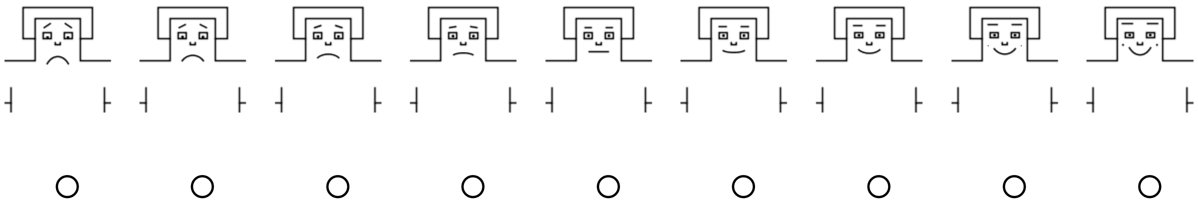

[Please indicate how agitated or aroused you have felt after reading the story. Try to answer **intuitively**. Please choose those figure, which best represents your current state of mind.]

How agitated do you feel right now?

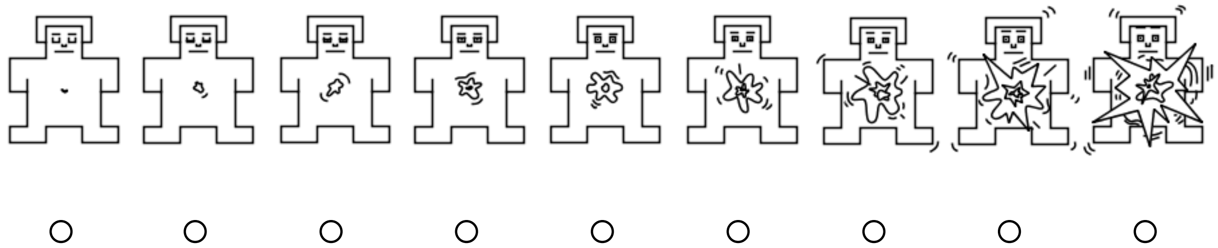

2.2 Rating of moral emotions

"When thinking about the protagonist's decision, I feel … towards the protagonist"

	complete-ly disa-gree **-3**	**-2**	**-1**	**0**	**+1**	**+2**	complete-ly agree **+3**
Elevation	○	○	○	○	○	○	○
Hopelessness	○	○	○	○	○	○	○
Gratitude	○	○	○	○	○	○	○
Disappointment	○	○	○	○	○	○	○
Disgust	○	○	○	○	○	○	○
Contempt	○	○	○	○	○	○	○
Anger / Indignation / Moral Out-rage	○	○	○	○	○	○	○
Compassion	○	○	○	○	○	○	○
Sympathy	○	○	○	○	○	○	○
Comprehension	○	○	○	○	○	○	○
Worry about society	○	○	○	○	○	○	○

2.3 Judging protagonists’ actions

Should the protagonist be punished for his/her actions? If yes, how harsh should the punishment be?

Please choose one of the following alternatives.

○ No punishment

○ Minor punishment (up to 36 months in prison)

○ Intermediate punishment (between 3 and 10 years in prison)

○ Major punishment (more than 10 years in prison)

How long should the protagonist be imprisoned?

10 years [slider] 100 years

Note: Depending on participants’ response to the filter question, anchors of the displayed scroll bar varied. Under the scrolling bar the selected imprisonment duration was displayed as numerical value.

2.4 Cognitive Ratings

Please rate the following statements regarding the scenario you just read.

	completely disagree<br>**-3**	**-2**	**-1**	**0**	**+1**	**+2**	completely agree<br>**+3**
I consider the decision of the protagonist appropriate.	○	○	○	○	○	○	○
While reading, I imagined the situation vividly.	○	○	○	○	○	○	○
I found it hard to decide which form of punishment is appropriate.	○	○	○	○	○	○	○

Supplementary Analyses

Decision Difficulty and Moral Emotions

We further investigated the outcome variables' interdependency. We calculated multilevel regression models with random intercepts for subjects using the R packages "lme4" (Bates, Mächler, Bolker, & Walker, 2015) and "lmerTest" (Kuznetsova, Brockhoff, & Christensen, 2016). We hypothesized that the difficulty to decide on an appropriate punishment might depend on conflicting emotional tendencies towards the perpetrator. Thus, observers should find decisions easy if negative *or* positive emotions, respectively, are clearly predominant. However, the more similar these different emotions are in strength, the harder it should be for observers to make a decision. We predicted decision difficulty as a quadratic function of emotional conflict (i.e., the difference of understanding and negative emotions). Across all seven scenarios with instrumental killings used in this study, the model, $F(1, 6797.71) = 935.85$, $p <$ .001, $R^2 = .324$, revealed that decision difficulty depends as an inverse parabolic (U-shaped) function on the balance of understanding and negative emotions, $b(SE) = -0.106\ (0.003)$, $\beta = -.33$, $t(6796.84) = -30.60$, $p < .001$, $\eta^2 = .129$ (Supplementary Figure 1)..

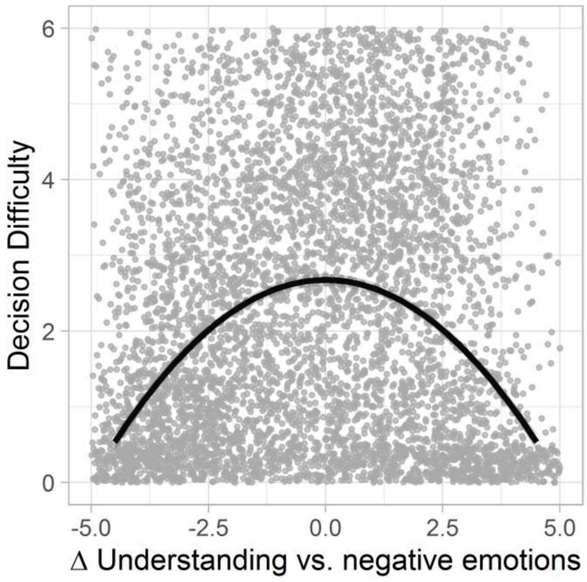

Supplementary Figure 1. Third-parties' difficulty to decide on an appropriate punishment depending on the difference in strength of positive and negative emotions, i.e., emotional conflict.

Post-hoc linear contrasts of the main analyses

Supplementary Table 1. Results of linear slope contrasts to characterize main and interaction effects of the main analyses (*N*=1004)

Personality factor	Outcome	Linear slope contrast	*b*	*SE*	p_{nom}	p_{Holm}
PF1	Negative emotions	Selfish killing of avoidable vs. inevitable victims	0.26	0.04	<.00001	<.00001***
	Moral appropriateness	Selfish vs. utilitarian killing	-0.06	0.04	.13196	.26393
PF3	Punishment	Selfish-inevitable vs. all other conditions	0.74	0.18	.00006	.00037**
	Negative emotions	Selfish vs. utilitarian killing	-0.03	0.02	.31541	.31541
	Negative emotions	Selfish killing of avoidable vs. inevitable victims	0.13	0.04	.00030	.00132**
	Understanding emotions	Selfish vs. utilitarian killing	0.29	0.04	<.00001	<.00001***
	Understanding emotions	Selfish killing of avoidable vs. inevitable victims	0.27	0.05	<.00001	<.00001***
	Moral appropriateness	Selfish vs. utilitarian killing	-0.22	0.04	<.00001	<.00001***
PF5	Punishment	Selfish vs. utilitarian killing	0.66	0.15	.00001	.00008***
	Punishment	Inevitable vs. avoidable victims	0.52	0.15	.00046	.00139**
	Negative emotions	Selfish vs. utilitarian killing	0.2	0.02	<.00001	<.00001***
	Negative emotions	Utilitarian killing of avoidable vs. inevitable victims	0.12	0.03	.00026	.00132**
	Understanding emotions	Selfish vs. utilitarian killing	0.33	0.03	<.00001	<.00001***
	Understanding emotions	Inevitable vs. avoidable victims	-0.24	0.03	<.00001	<.00001***
	Understanding emotions	Utilitarian killing of avoidable vs. inevitable victims	-0.33	0.05	<.00001	<.00001***
	Moral appropriateness	Selfish vs. utilitarian killing	0.15	0.03	.00001	.00008***

Note: * $p < .0125$, ** $p < .0025$, *** $p < .00025$, two-tailed, according to the Bonferroni-correction applied to the main analyses.

Additional Contrast Analyses I: *Footbridge* vs. *Transplant*

As outlined in the Method section, we contrasted responses to additional dilemmas that featured instrumental killings to test whether our original results held up in different moral scenarios. First, we contrasted *footbridge* and *transplant*. In both dilemmas, the protagonist decides to kill one person in order to save five others. However, the scenarios differ as to whether or not the protagonist experiences an additional role conflict. While in *footbridge*, the perpetrator is a stranger out for a walk with no relationship to the victim, in *transplant,* the perpetrator is the victim's physician and thus under the professional obligation to do no harm. A potentially confounding factor is the difference in method of killing.

Contrast analyses showed that these content differences had a considerable effect on observers' responses. The physician in *transplant* received an average prison sentence that was 19.5 years longer than the perpetrator's sentence in *footbridge*. Correspondingly, *transplant* elicited more marked negative emotions and less understanding sentiments towards the perpetrator in observers. In addition, observers rated the actions in *transplant* as less morally appropriate than in *footbridge* (all p's < .001).

As in the main analysis, differences in the personality traits comprising PF1 (empathy/altruism), PF3 (trait psychopathy) and PF5 (intuitive/authority-obedient thinking) were linked to differences in observers' responses. Contrariwise, PF2 (anxiety, personal distress) and PF4 (justice sensitivity) showed no significant associations after Bonferroni correction.

In detail, **PF1** (empathy/altruism) was linked to increased negative emotions in both scenarios, but particularly to heightened negative emotions towards the perpetrator in *footbridge*. However, PF1 was also linked to reduced moral appropriateness ratings in the *footbridge* scenario.

Higher **PF3** (trait psychopathy) scores were associated to (i) harsher punishments in both *transplant* and *footbridge*; (ii) slightly less negative emotions towards the perpetrator in *transplant*; (iii) increased perceived moral appropriateness in *footbridge*, but not *transplant*.

PF5 (intuitive/authority-obedient thinking) was linked to (i) harsher punishments in both scenarios; (ii) increased negative emotions, particularly in *footbridge*; (iii) specifically decreased understanding emotions in *footbridge* in individuals with high PF5 scores and specif-

ically heightened understanding emotions in footbridge in observes with low PF5 scores (i.e., individuals with high NFC). Thus, individuals with high PF5 scores (intuitive/authority-obedient thinking) punished utilitarian killings more harshly in general and felt more intense negative emotions towards the perpetrators. Furthermore, while differences in PF5 were linked to differences in understanding emotions in *footbridge*, this was not the case in *transplant*. In sum, individuals with tendencies towards intuitive/authority-obedient thinking show less understanding in general for utilitarian killings. In addition, particularly in the absence of a role conflict (*footbridge*) differences in PF5 resulted in large differences in understanding sentiments (see Supplementary Figure 2).

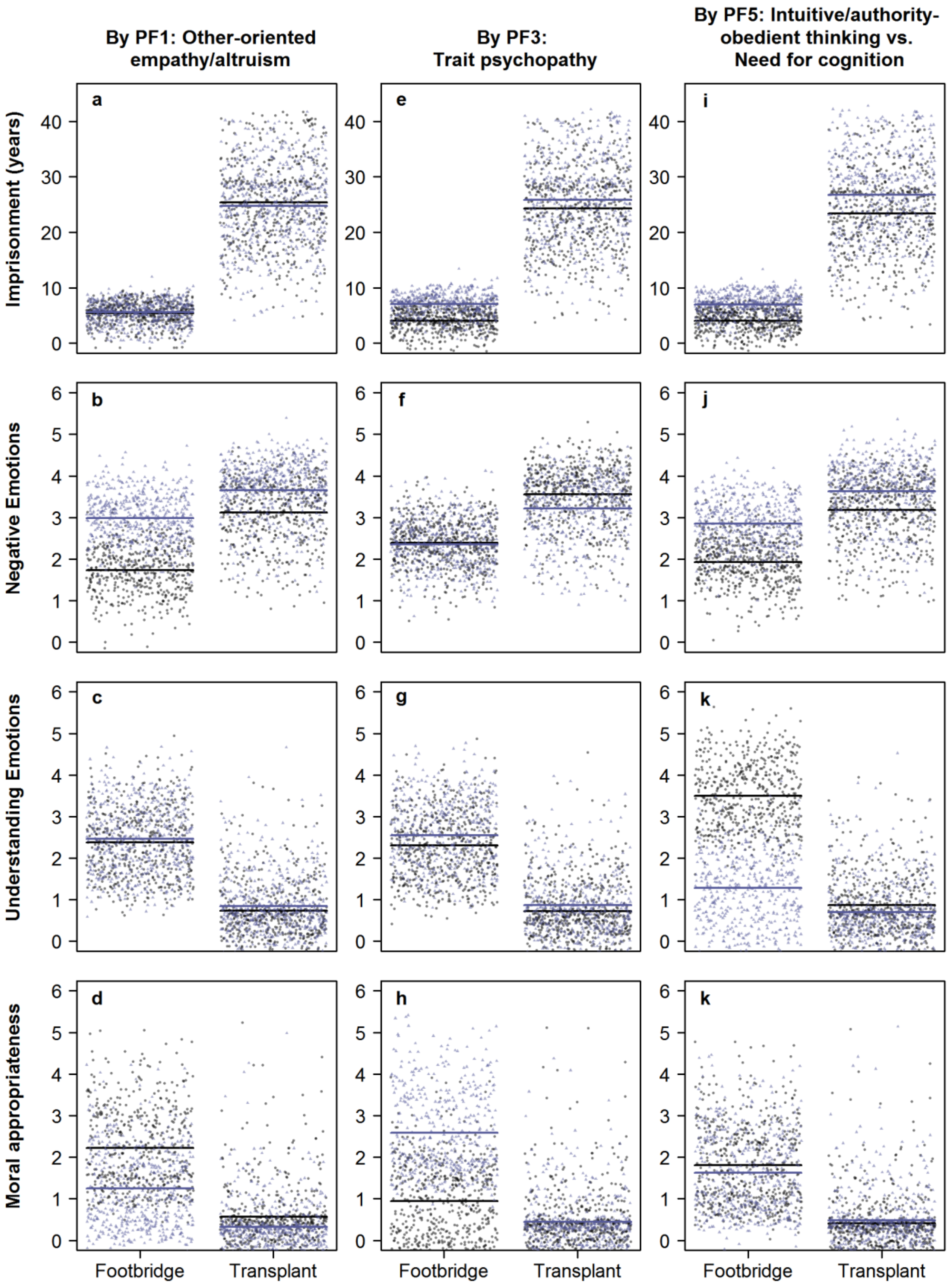

Supplementary Figure 2. Personality-dependent differences in observers' responses to utilitarian killings (*transplant* and *footbridge*). Lower (*M*-2*SD*) and higher values (*M*+2*SD*) of the respective personality domain are indicated by black and light grey color, respectively.

Supplementary Table 2. Results of linear mixed effect models contrasting *transplant* and *footbridge* (reference scenario) (*N*=1004)

	**Imprisonment duration (years)**						**Negative Emotions [0,6]**						**Understanding Emotions [0,6]**						**Perceived moral appropriateness [0,6]**					
Model factors	*b*	*SE*	β	*t*	*p*	η^2_p	*b*	*SE*	β	*t*	*p*	η^2_p	*b*	*SE*	β	*t*	*p*	η^2_p	*b*	*SE*	β	*t*	*p*	η^2_p
Intercept	5.58	0.22		25.43	**<.001**		2.38	0.03		70.52	**<.001**		2.39	0.04		66.29	**<.001**		1.77	0.04		49.18	**<.001**	
Scenario	19.47	0.27	.81	70.93	**<.001**	.75	1.00	0.04	.41	27.88	**<.001**	.36	-1.59	0.04	-.55	-39.08	**<.001**	.51	-1.32	0.04	-.48	-30.48	**<.001**	.37
PF1	0.05	0.26	.00	0.21	.835	.00	0.31	0.04	.25	7.96	**<.001**	.03	0.02	0.04	.02	0.53	.598	.00	-0.24	0.04	-.18	-5.80	**<.001**	.01
PF2	-0.08	0.23	-.01	-0.34	.731	.00	0.09	0.04	.07	2.41	**.016**	.00	-0.03	0.04	-.02	-0.74	.459	.00	0.00	0.04	.00	-0.09	.932	.00
PF3	0.74	0.24	.06	3.06	**.002**	.01	-0.01	0.04	-.01	-0.24	.811	.00	0.06	0.04	.04	1.61	.108	.00	0.41	0.04	.30	10.29	**<.001**	.03
PF4	-0.02	0.25	.00	-0.06	.951	.00	0.07	0.04	.05	1.72	.085	.01	0.04	0.04	.03	1.00	.316	.00	0.02	0.04	.02	0.53	.595	.00
PF5	0.72	0.23	.06	3.20	**.001**	.01	0.23	0.03	.19	6.63	**<.001**	.02	-0.55	0.04	-.38	-14.97	**<.001**	.06	-0.04	0.04	-.03	-1.18	.237	.00
Scenario × PF1	-0.22	0.32	-.01	-0.68	.496	.00	-0.18	0.04	-.10	-4.27	**<.001**	.01	0.01	0.05	.00	0.11	.912	.00	0.18	0.05	.09	3.61	**<.001**	.01
Scenario × PF2	-0.33	0.29	-.02	-1.13	.257	.00	-0.07	0.04	-.04	-1.85	.064	.00	0.07	0.04	.03	1.65	.099	.00	0.04	0.05	.02	0.77	.440	.00
Scenario × PF3	-0.37	0.30	-.02	-1.21	.227	.00	-0.08	0.04	-.04	-1.95	.051	.00	-0.03	0.04	-.01	-0.62	.538	.00	-0.41	0.05	-.21	-8.62	**<.001**	.05
Scenario × PF4	0.56	0.31	.03	1.82	.070	.00	0.04	0.04	.02	1.02	.307	.00	-0.07	0.05	-.04	-1.61	.108	.00	-0.02	0.05	-.01	-0.43	.667	.00
Scenario × PF5	0.11	0.28	.01	0.38	.701	.00	-0.12	0.04	-.07	-3.19	**.001**	.01	0.51	0.04	.25	12.22	**<.001**	.09	0.06	0.04	.03	1.37	.172	.00
Overall model statistic	$F(11, 1714.44) = 460.83$, $p < 2.2e\text{-}16^{***}$, $R^2 = .738$						$F(11, 1714.44) = 86.96$, $p < 2.2e\text{-}16^{***}$, $R^2 = .573$						$F(11, 1714.44) = 162.23$, $p < 2.2e\text{-}16^{***}$, $R^2 = .602$						$F(11, 1714.44) = 105.85$, $p < 2.2e\text{-}16^{***}$, $R^2 = .501$					

Additional Contrast Analyses II: *Crying Baby* vs. *Sophie's Choice*

The second contrast focused on utilitarian killings of children. In both scenarios, the respective mothers decide to either suffocate their own baby (*crying baby*) or hand over their child to killers (*Sophie's choice*) to protect the life of others. The scenarios contrast personal (*crying baby*) vs. impersonal killing (*Sophie's choice*). However, the also differ with regard to additional content features and thus potentially confounding factors, e.g., type of kin saved (husband vs. other child) and additional saving of other people.

On average, observers punished the perpetrator in *crying baby* more harshly. However, in both scenarios the majority of participants decided *not* to punish at all (*crying baby*: 69.2% vs. *Sophie's choice*: 92.8%; McNemar test on paired proportions: Δ= 23.6%, $p < .001$). Negative emotions did not differ between the scenarios. However, understanding emotions and moral appropriateness ratings were increased in *Sophie's choice* compared to *crying baby*.

As for personality, differences in PF1 (empathy/altruism), PF3 (trait psychopathy) and PF5 (intuitive/authority-obedient thinking) showed association with participants' responses (see supplementary figure 3). In contrast, PF2 (anxiety, personal distress) and PF4 (justice sensitivity) again showed either no associations or had only marginal effects that did not survive Bonferroni correction.

Analyses revealed main effects of **PF1** (empathy/altruism) on negative and understanding emotions. In *crying baby* as well as *Sophie's choice*, both types of emotions were reported to be more intense by observers with higher PF1 scores. Thus, more empathic/altruistic individuals respond with more intense moral emotions of any kind to the utilitarian killing of children as described in the two dilemmas, and probably experience as a result a heightened emotional conflict.

PF3 (trait psychopathy) was associated with (i) slightly harsher punishments in *Sophie's choice*; (ii) increased understanding emotions for the mother in *crying baby* and but less understanding for the mother in *Sophie's choice*; (iii) increased moral appropriateness ratings specifically in *crying baby.*

Finally, higher scores in **PF5** (intuitive/authority-obedient thinking) were linked across both scenarios to (i) harsher punishment; (ii) increased negative emotions; (iii) reduced understanding emotions; and (iv) reduced moral appropriateness ratings. Thus, individuals with a higher tendency towards intuitive/authority-obedient thinking consider utilitarian killings of children in general to be more punishable and less morally appropriate. Correspondingly they report increased negative and reduced understanding moral emotions towards perpetrators.

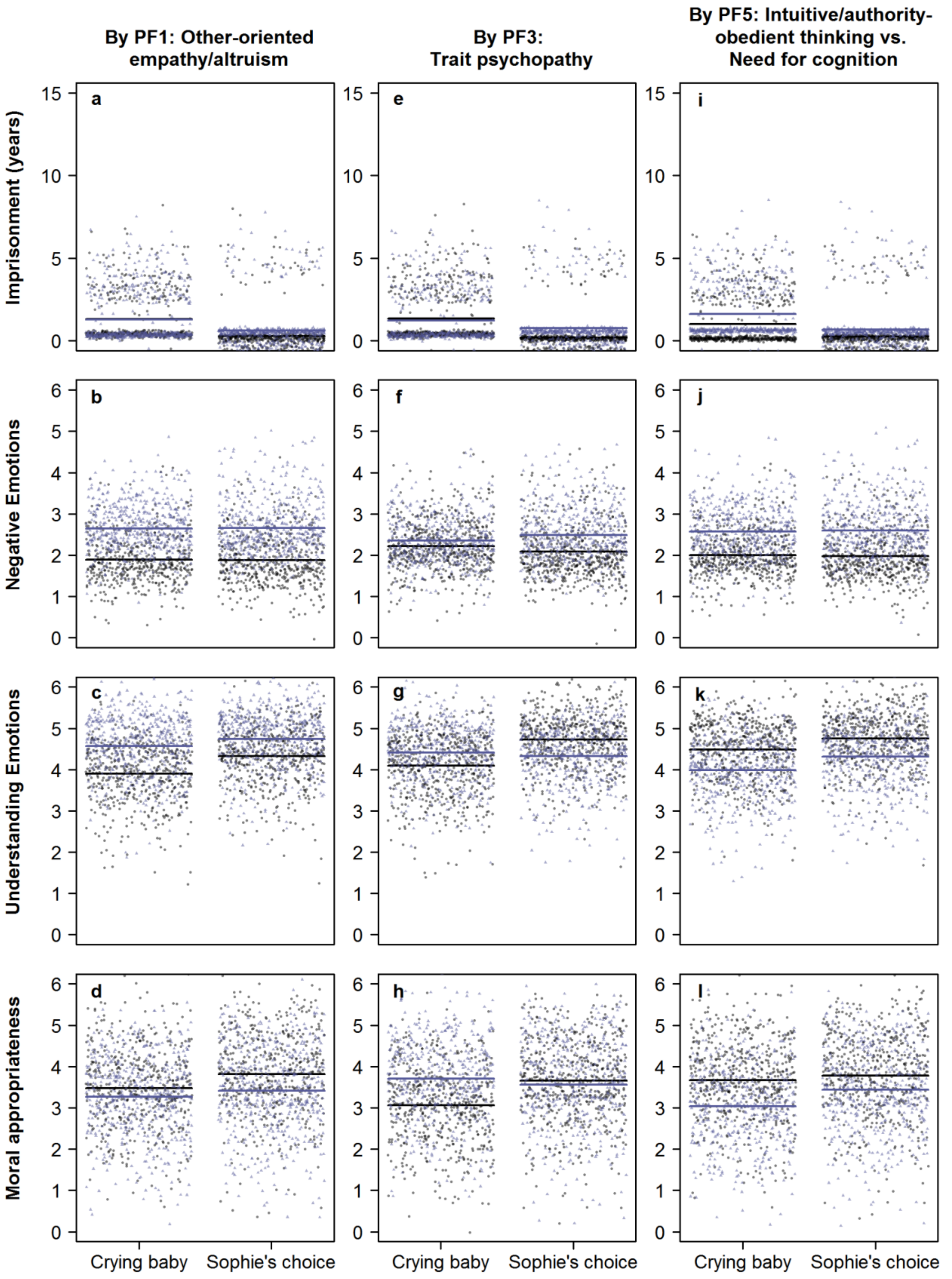

Supplementary Figure 3. Personality-dependent differences in observers' responses to utilitarian killings of children (*Crying baby* and *Sophie's choice*). Lower (*M*-2*SD*) and higher values (*M*+2*SD*) of the respective personality domain are indicated by black and light grey color, respectively.

Supplementary Table 3. Results of linear mixed effect models contrasting *crying baby* and *Sophie's choice* (*N*=1004)

	**Imprisonment duration (years)**						**Negative Emotions [0,6]**						**Understanding Emotions [0,6]**						**Perceived moral appropriateness [0,6]**					
Model factors	*b*	*SE*	β	*t*	*p*	η^2_p	*b*	*SE*	β	*t*	*p*	η^2_p	*b*	*SE*	β	*t*	*p*	η^2_p	*b*	*SE*	β	*t*	*p*	η^2_p
Intercept	1.29	0.06		21.33	**<.001**		2.28	0.04		64.72	**<.001**		4.23	0.04		109.52	**<.001**		3.38	0.05		70.94	**<.001**	
Scenario	-0.82	0.07	-.21	-11.09	**<.001**	.07	0.01	0.03	.01	0.40	.690	.00	0.28	0.04	.11	7.10	**<.001**	.04	0.22	0.05	.07	4.24	**<.001**	.01
PF1	-0.01	0.07	.00	-0.13	.896	.00	0.19	0.04	.17	4.61	**<.001**	.02	0.17	0.05	.13	3.71	**<.001**	.01	-0.05	0.06	-.03	-0.93	.352	.00
PF2	-0.06	0.06	-.03	-0.86	.390	.00	-0.03	0.04	-.03	-0.92	.360	.00	0.00	0.04	.00	0.04	.970	.00	0.07	0.05	.04	1.35	.177	.00
PF3	-0.03	0.07	-.02	-0.50	.620	.00	0.03	0.04	.03	0.87	.382	.00	0.08	0.04	.07	1.92	**.055**	.00	0.16	0.05	.11	3.11	**.002**	.00
PF4	0.00	0.07	.00	0.06	.953	.00	0.03	0.04	.02	0.64	.524	.00	0.03	0.04	.02	0.69	.492	.00	-0.03	0.05	-.02	-0.55	.582	.00
PF5	0.15	0.06	.08	2.46	**.014**	.00	0.14	0.04	.13	3.99	**<.001**	.02	-0.12	0.04	-.10	-3.08	**.002**	.01	-0.16	0.05	-.10	-3.24	**.001**	.01
Scenario × PF1	0.10	0.09	.04	1.16	.247	.00	0.01	0.04	.00	0.18	.853	.00	-0.06	0.05	-.03	-1.34	.179	.00	-0.05	0.06	-.02	-0.78	.436	.00
Scenario × PF2	0.10	0.08	.04	1.33	.182	.00	0.09	0.04	.06	2.50	**.013**	.00	-0.01	0.04	.00	-0.13	.897	.00	-0.11	0.06	-.05	-1.94	**.053**	.00
Scenario × PF3	0.18	0.08	.06	2.19	**.029**	.00	0.07	0.04	.04	1.76	.078	.00	-0.18	0.04	-.10	-4.17	**<.001**	.01	-0.19	0.06	-.09	-3.21	**.001**	.01
Scenario × PF4	-0.09	0.08	-.03	-1.13	.257	.00	0.03	0.04	.02	0.72	.470	.00	0.01	0.04	.00	0.15	.877	.00	0.01	0.06	.00	0.16	.873	.00
Scenario × PF5	-0.05	0.08	-.02	-0.63	.527	.00	0.01	0.04	.01	0.25	.804	.00	0.01	0.04	.01	0.32	.752	.00	0.07	0.05	.03	1.35	.178	.00
Overall model statistic	$F(11, 1714.44) = 12.71$, $p < 2.2e{-16}^{***}$, $R^2 = .298$						$F(11, 1714.44) = 7.24$, $p < 2.2e{-16}^{***}$, $R^2 = .548$						$F(11, 1714.44) = 9.20$, $p < 2.2e{-16}^{***}$, $R^2 = .602$						$F(11, 1714.44) = 4.66$, $p < 2.2e{-16}^{***}$, $R^2 = .405$					
